# Changing landscape configuration demands ecological planning: Retrospect and prospect for megaherbivores of North Bengal

**DOI:** 10.1371/journal.pone.0225398

**Published:** 2019-12-19

**Authors:** Tanoy Mukherjee, Lalit Kumar Sharma, Mukesh Thakur, Goutam Kumar Saha, Kailash Chandra

**Affiliations:** 1 Zoological Survey of India, Prani Vigyan Bhawan, New Alipore, Kolkata, West Bengal, India; 2 Department of Zoology, University of Calcutta, Ballygunge, Kolkata, West Bengal, India; Centre for Cellular and Molecular Biology, INDIA

## Abstract

The Gorumara National Park (GNP) is an important conservation area located in the northern region of West Bengal State, India, as it provides habitat for three megaherbivores: Indian One-horned rhinoceros (*Rhinoceros unicornis*), Asian elephants (*Elephas maximus*) and Gaurs (*Bos gaurus*). It harbours one of the last population of the one-horned rhino. In the present study, landscape change and configuration were investigated by comparing three Landsat images, from 1998, 2008 and 2018. The images were classified into six different landcover classes following standard methodology. The present study also involves evaluation of landscape and anthropogenic predictors influence on the megaherbivores of GNP, followed by future landcover simulation for the year 2028. The result shows a significant decrease in the grassland cover from 18.87 km^2^ to 8.27 km^2^ from 1998 to 2018, whereas the woodland cover has increased from 50.14 km^2^ to 62.09 km^2^ between 1998 and 2018. The landscape configuration indices such as Number of Patches (NP), Patch Density (PD), Interspersion and Juxtaposition (IJI), Aggregation Index (AI) and Mean Shape Index (SHAPE AM) indicated that the landscapes has lost complexity in the spatial placement of patches of different Land Use and Land Cover (LULC) classes. Also, the landscape over the three decades has become uniform in terms of diversity of patches, because of earlier plantation activities by the forest managers. Result also indicated that grassland, along with its class metrics are the top predictors contributing 43.6% in explaining the spatial distribution of megaherbivores in GNP. Results from the simulated landcover of 2028 suggest a possible decline in overall grassland by 6.23% and a subsequent upsurge in woodland by 6.09% from 2018. The present result will be useful in guiding the forest management in developing habitat improvement strategies for the long- term viability of megaherbivore populations of rhino, gaur and elephant in the GNP.

## Introduction

Detecting the rate of change and pattern of change in the landscape has been considered as an essential theme in ecological research [1, 2]. Multi-temporal change analysis of remotely sensed data enables us to understand the dynamicity in landscape composition [3,4,5,6]. Understanding the pattern of change in the landscape configuration of Protected Areas (PAs) including National Parks (NP), Wildlife Sanctuaries (WLS) and reserves would eventually help the managers making strategies for effective conservation and management. A number of studies are available which have demonstrated the applicability of mapping and landscape change analysis for long-term conservation and management [7,8,9,10,11].

Megaherbivores (mammals with bodyweight over 10^3^ kg) generally need more food and space than mesoherbivores, and these two, being the common constraints in areas with high population pressure as there population regulation through predation processes is very limited [12]. In today’s world, most productive habitats like floodplains and foothills are the areas where the megaherbivores are generally found. However, such regions are profoundly affected by habitat loss, fragmentation and other anthropogenic disturbances, restricting their population into small pockets [12,13]. Moreover, megaherbivores are known to have powerful effects on both the assembly and composition of vegetation and hence play an essential role in ecosystem dynamics [14].

Gorumara National Park (GNP) is known for its large assemblage of megaherbivores i.e. Indian One-horned rhinoceros (*Rhinoceros unicornis*), Asian elephants (*Elephas maximus*) and Gaurs (*Bos gaurus*). The habitat stretch of (GNP) includes territorial forests of Jalpaiguri, Kalimpong and Cooch Behar Forest Divisions also Jaldapara Wildlife Sanctuary, Chapramari Wildlife Sanctuary. But the total stretch is not continuous, recently due to enormous expansion of tourism, roads and railways with other anthropogenic activities resulted in substantial degradation and fragmentation of the habitat stretch [15]. These activities may lead to a loss in both movement corridors as well as genetic vigour of the wildlife populations [16,17].

Despite various threats in the past decades, the park management could be able to reduce the poaching threat successfully, that has resulted in a considerable increase in rhino population, i.e. 50 individuals [18]. However, due to massive population expansion of other sympatric megaherbivores, mostly *Bos gaurus* with over 900 individuals and *Elephas maximus* in GNP have resulted in an ecological crisis and have led to increased interspecific competitions [15]. For this reason, rhinos are frequently found outside its natural habitat (far-flung as 50 km) to meet their foraging needs, and some of them are staying seasonally or even permanently [19].

Importance of grasses and grassland, both on their qualitative and quantitative aspects is deeply associated with megaherbivores of GNP. For Rhinos, they were found to prefer grasslands intermixed with wetland and riverine forests [20]. Studies also indicate that grasses are the most preferred diet for rhino [21,22,23]. Similar types of findings were also observed across most Indian one-horned rhino reserves from India and Nepal [24,25]. Hence it can be opined that the rhinos are more habitat specialised than other megaherbivores and depend on food quality rather than quantity [22,26].Moreover, in Indian subcontinent gaurs are primarily grazers, and their diet is largely composed of grasses. The preference of grasses by gaur is supported by findings from various studies throughout Indian subcontinent [27–31]. On the other hand, elephants are known to exploit a large variety of species, yet the dominance of grasses in the diet of the elephant was documented by various studies, including GNP [32–35].

However, among the many ecosystems of GNP, grassland are the most affected ones in recent times, which is evident by a sharp decline in both productivity and species diversity. Leading causes of which include overgrazing, soil erosion, nutrient depletion, salinization, pollution, disruption of hydrological systems, and conversion of natural areas into croplands, monoculture plantations and ill-planned developmental activities resulting in exceeded carrying capacity for rhino [19]. The carrying capacity for rhino in Gorumara was first assessed to be 20 in the year 1995 [36]. Followed by several other studies which suggest they exceeded carrying capacity threshold for rhino in GNP [19,37].

Landscape-level characterization remains associated with a qualitative and quantitative evaluation which helps in identifying the linkage between spatial pattern and ecological processes, like composition, configuration and connectivity [16,38,39]. Landscape composition mainly refers to the number of different elements, their spatial distribution, relative abundance and diversity, while landscape configuration refers to physical orientation, heterogeneity, and distribution within the landscape [39]. Globally, forested habitats, including forests in protected areas, are facing severe management challenges posed by various planned and unplanned degradation drivers [40,41].

Spatial heterogeneity of these landscapes influences the distribution, abundance and population dynamics of species [42]. As a consequence, new ecological theories along with new applications for planning and monitoring have been developed. Studies have also been conducted towards identification and evaluation of drivers which greatly influencing the spatial distribution and habitat suitability of the species [43,44,45]. Moreover, several researchers have also used landscape metrics and presented their imperativeness in developing a better understanding of the landscapes which species are utilizing [45,46,47]. Most prominently indices about patch structure, their spatial placement and configuration in the landscape are useful in understanding the ecology of large mammalian species including elephants, rhinos [48–51].

Furthermore, recent techniques in remote sensing have provided opportunities for developing models for further understanding of the future landcover dynamics, which will eventually help in making management decisions [52]. Among various approaches for landcover modelling and predicting land use in future scenarios, Markov chain analysis has been used most commonly [53]. Markov chain analysis is based on a stochastic approach which predicts a specific state of an extensive system at a particular temporal scale based on the state of past trends by using different topographic and landcover drivers [54,55].

Hence, the present study has been designed by combining Land Use and Land Cover (LULC) analysis of georeferenced satellite data of three decades (1998, 2008 and 2018) with landscape ecology theories and methods. Our overall goal was to generate a better understanding of LULC change patterns and configuration of the GNP landscape, which is facing tremendous pressure because of recent human colonization and related development. We have also evaluated the influence of landscape and anthropogenic predictors on the spatial distribution of megaherbivores in GNP. Further, to understand the future scenario, we have used Markov-cellular automata (CA) technique based Artificial Neural Network model (ANN) for Spatio-temporal modelling of future landcover for the year 2028.

We seek to develop baselines for a variety of landscape indices, which can describe the conversion of formerly contiguous habitats and its impacts on animals with relatively large territories including rhinos, elephants and gaur [56–58]. The Indian one-horned rhino is the flagship and also the top conservation priority species of GNP, and most of the management strategies of the park are mostly concentrated around this species. Our analysis of three decades of landscape change will provide a retrospective view on landscape change and will help in planning long-term conservation measures for the megaherbivore populations.

## Material and methods

### Study area

**Fig 1:**
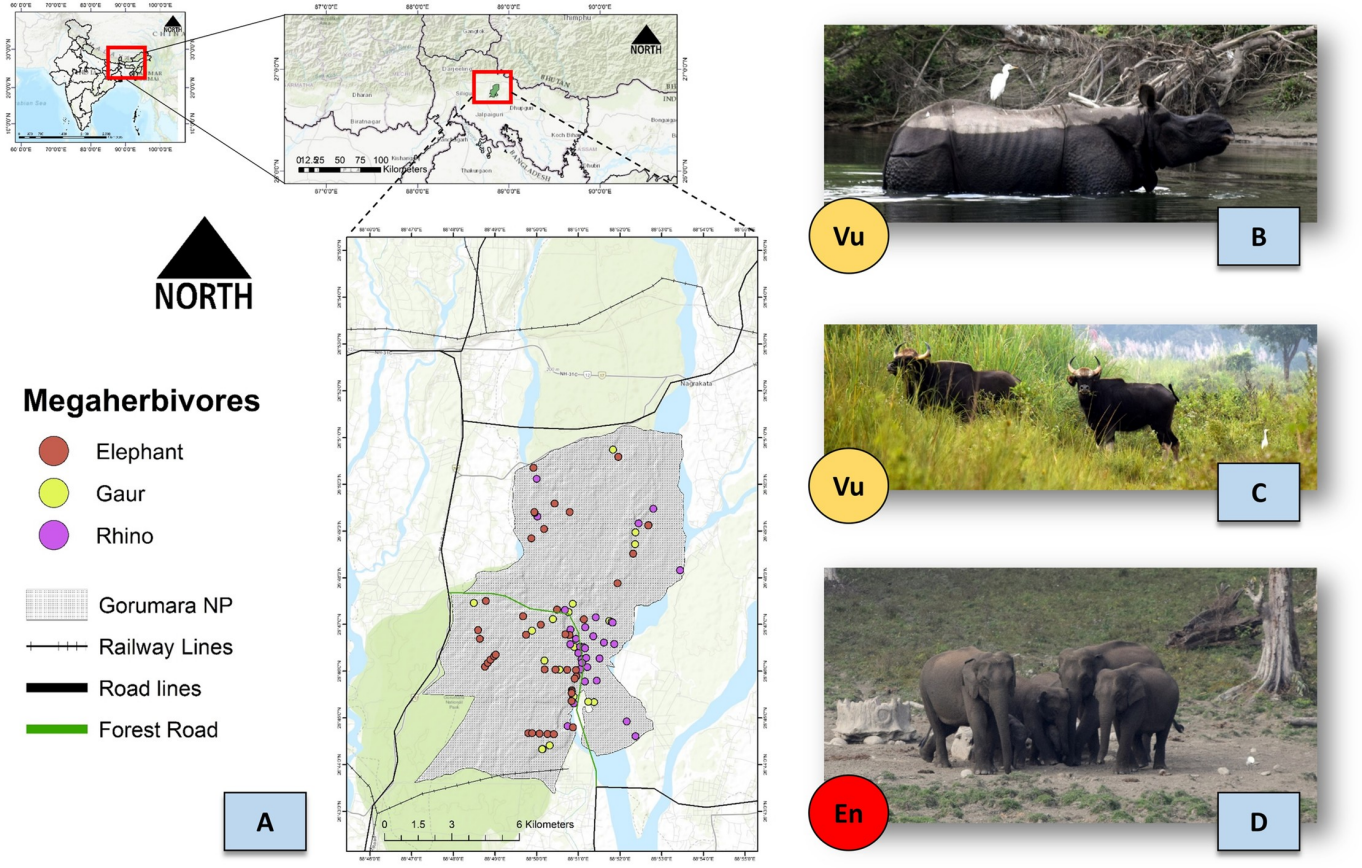
Study area map. A. Gorumara National Park (GNP), with road, rail networks and occurrence records of megaherbivores, i.e. Elephant, Gaur and Rhino. Topographic shading uses an ESRI base map (Topographic) in ArcGIS 10.6. B. Field picture of one-horned rhino (*Rhinoceros unicornis*) in its natural habitat of GNP. C. Field picture of Indian Gaur (*Bos gaurus*) in its natural habitat of GNP. D. Field picture of Indian Elephant (*Elephas maximus indicus*) in its natural habitat of GNP. Vu = Vulnerable and En = Endangered, representing the respective IUCN Redlist categories.

The GNP is located in the northern region of the West Bengal state of India (Fig 1), falling in the 7B-Lower Gangetic plan biogeographic zone situated between latitude 26°47’12.5” North to 26°43’25.6” North and longitude 88°52’04.2” East to 88°47’07.3” (Fig 1), with an area of 90 km^2^ [59]. The park is located in the foothills of the eastern Himalayas, popularly known as Dooars, composed of flood plains, hills, forests, grasslands and plantations. The landscape is known for its rich biodiversity which includes 193 species of birds, 22 species of reptiles, 40 species of fishes and other macro and micro fauna [60,61]. The rich diversity of herbivores is represented by Indian Rhinoceros, Gaur, Asian Elephant, Chital, Sambar, Barking deer, Hog deer and Wild boar. Common Leopard is the only large carnivore residing in the study area.

The terrain of the landscape is differentiated into distinct plateaus and lacks close contours. The river system includes three main seasonal rivers, namely, the Murti, Indong and Garati Rivers, which drain into the Jaldakha River. The vegetation in the landscape is dominated by the tree species *Shorea robusta*, *Lagerstroemia speciosa*, *Senegalia catechu* and *Dalbergia sissoo*, and classified as riverine forests, sal forests, wet mixed forests and sal savannah [62]. The GNP contains a mosaic of mixed land-use and land-cover types in which human-wildlife conflict management is a major challenge for the authorities as well a source of threat to the large mammals in the study area [63].

Earlier forested habitat of GNP was a part of Jalpaiguri Forest division comprising of an area only 8.62 km^2^ and the area was declared as Gorumara Game Sanctuary vide Govt. Notification No. 5181-For, Dt: 02.08.1949 just after independence. Afterwards through subsequent notification finally it got the status of National Park in the year 1998 [62,63]. Before 1994, the primary aim of this forest areas was to maximise timber production through the plantation of commercial species, such as Sal (*Shorea robusta*), Teak (*Tectona grandis*) and Jarul (*Lagerstroemia speciosa*). Hence, the forest continues to be exploited under various working plans from 1892 onwards until the end of the 4^th^ working plan (1926–27 to 1945–46). However, after the notification of the area as National Park, the objective has changed from production forestry to protection, for the conservation of wildlife and biodiversity. The first Management Plan of GNP was prepared and approved by Ministry of Environment and Forests, Govt. of India in 1997–98 with the aim of habitat improvement with focus on flagship species rhinoceros and other wild animals. As a habitat improvement strategy, canopy opening was done till year 2000, and then it has been stopped due to ban imposed by the Supreme Court of India. Later in the year 2006, the ban was lifted by the apex court for limited activities, but no further canopy opening initiative has been documented in the recent management plan [62,63]. Presently, the management objectives in GNP include conservation of biodiversity with special thrust on the rhino, development of GNP as elephant reserve, community participation in forest management and promote ecologically sustainable tourism for nature education and awareness creation.

### Study design and data collection

For understanding the LULC change in the study area, we used three sets of Landsat satellite data (1998, 2008 and 2018), downloaded from the online USGS postal (https://earthexplorer.usgs.gov). For the landcover-change analysis, we used two types of Landsat sensor data. For 1998 and 2008, we used Landsat- 5 TM (Thematic Mapper), and for 2018 we used Landsat- 8 OLI (Operational Land Imager). All images were scaled for a spatial resolution of 30 m. To address the change in sensors from Landsat-5 TM and Landsat-8 OLI sensors, we used DEM data and geo-referenced the respective Landsat images following the robust method suggested by [28,29]. All images were projected in WGS_1984_UTM_Zone_45N (Table 1). Atmospheric, along with DOS1, corrections have been done for all three images using the semi-automatic classification tool for QGIS [64] followed by ML (Maximum Likelihood) technique for classification. All three images were classified into six major LULC classes: Woodland, Grassland, Shrubland, Bareland, Riverbank and Water. For the present study we have applied similar classification approach for the classification of 1998 and 2008 images by overlying training data polygon of 2018 on both 1998 and 2008 images followed by selection and elimination process for training samples which showed change in the cover type [65–67]. A total of 137 polygons comprising 973 pixels were taken during the field survey and were used on all three decadal images (1998, 2008 and 2018). Finally, we assessed the accuracy of the classified image by estimating the error matrix along with the kappa statistics. Sample points for accuracy estimation were also collected during the field visits; at least 60 samples location were taken for each land cover type for the present land cover.

**Table 1:** Information regarding satellite images.

Sensor	Path/row	Acquisition date
Landsat 5 Thematic Mapper (TM)	Path:139 Row:41	29-DEC-98
Landsat 5 Thematic Mapper (TM)		08-DEC-08
Landsat 8 Operational Land Imager (OLI)		20-DEC-18

Furthermore, Google Earth images and Forest Management Inventory data have been used for the past decadal images [68,69]. We calculated transition probabilities to determine the probability of transition from one land-cover type to another [68]. All image related analysis, as well as accuracy assessments, were carried out using the semi-automatic Classification plugin of QGIS, ENVI 5.1 and ESRI ArcGIS 10. 6.

We used the FRAGSTATS 4.2 program [70] to calculate a variety of landscape indices (S1 Table) for characterizing changes in land cover and landscape configuration across the three-decade images [71,72]. All the maps including the LULC, landscape configuration indices such as diversity index, patch density and patch density richness were developed using the ArcGIS 10.6 software program. The LULC and the landscape configuration change were assessed by comparing the three decadal data and by estimating the transition probability matrices between 1998 and 2008; 2008 to 2018 of the respective multi-temporal images using a Markovian approach.

### Evaluation of landscape and class predictors influence on the Megaherbivores of GNP

Predictor inspection was performed by using Generalized Additive Model (GAM) [73] in SAHM package of VistTrails environment [73,74]. For understanding the relative response to landscape configuration, topographic as well as anthropogenic predictors by the mega-herbivores of GNP the GAM was chosen for its adaptability in dealing with a non-linear relationship between response and explanatory variables [75]. The land cover classes and linear features (Road and Rail) variables were prepared by converting raster into vector followed by generating euclidian distance, whereas, landscape and class level metrics were prepared by using the moving window function of FRAGSTAT Ver. 4.2 (S4 Fig).

A total of n = 97 spatially independent GPS locations of direct sightings as well as indirect signs for all three mega-herbivores (Elephant, Rhino and Gaur) were collected by surveying line transects marked systematically in the landscape after stratification of habitat types in GNP between the year 2016 to 2018 (Fig 1). For evaluating relative responses of different variables, pseudoabsence points have been assessed by generating random points in ArcGIS 10.6 in the entire landscape. For all presence records the GPS location, habitat type, distance to water and distance to rail and road have also been recorded.

### Landcover simulation for the year 2028

We used Artificial Neural Network (ANN) based model for generating a future land cover map for the year 2028 of GNP in MOLUSCE plugin of QGIS using the classified images of 2008 and 2018 [68]. Furthermore, for simulating the landscape cellular-automata method based on Monte Carlo algorithm was used [68,76,77,78]. A total of five uncorrelated variables with Pearson’s correlation threshold less than 0.8 were selected as predictor variables for the simulation, and all the rasters have been resampled in 30 m cell size. The topographic variables, i.e. Compound Topographic Index [79,80], linear aspect and roughness, were used as surface texture/configuration drivers. Moreover, temperature and moisture drivers, i.e. Heat Load Index [81] and Integrated Moisture Index [82] were kept as a proxy for climatic drivers. All raster images were prepared using Geomorphometric and Gradient Metrics Toolbox Ver. 2.0 [83]. The validation of the simulated landcover of 2028 was carried out using percentage correctness along with kappa statistics.

## Results

**Fig 2:**
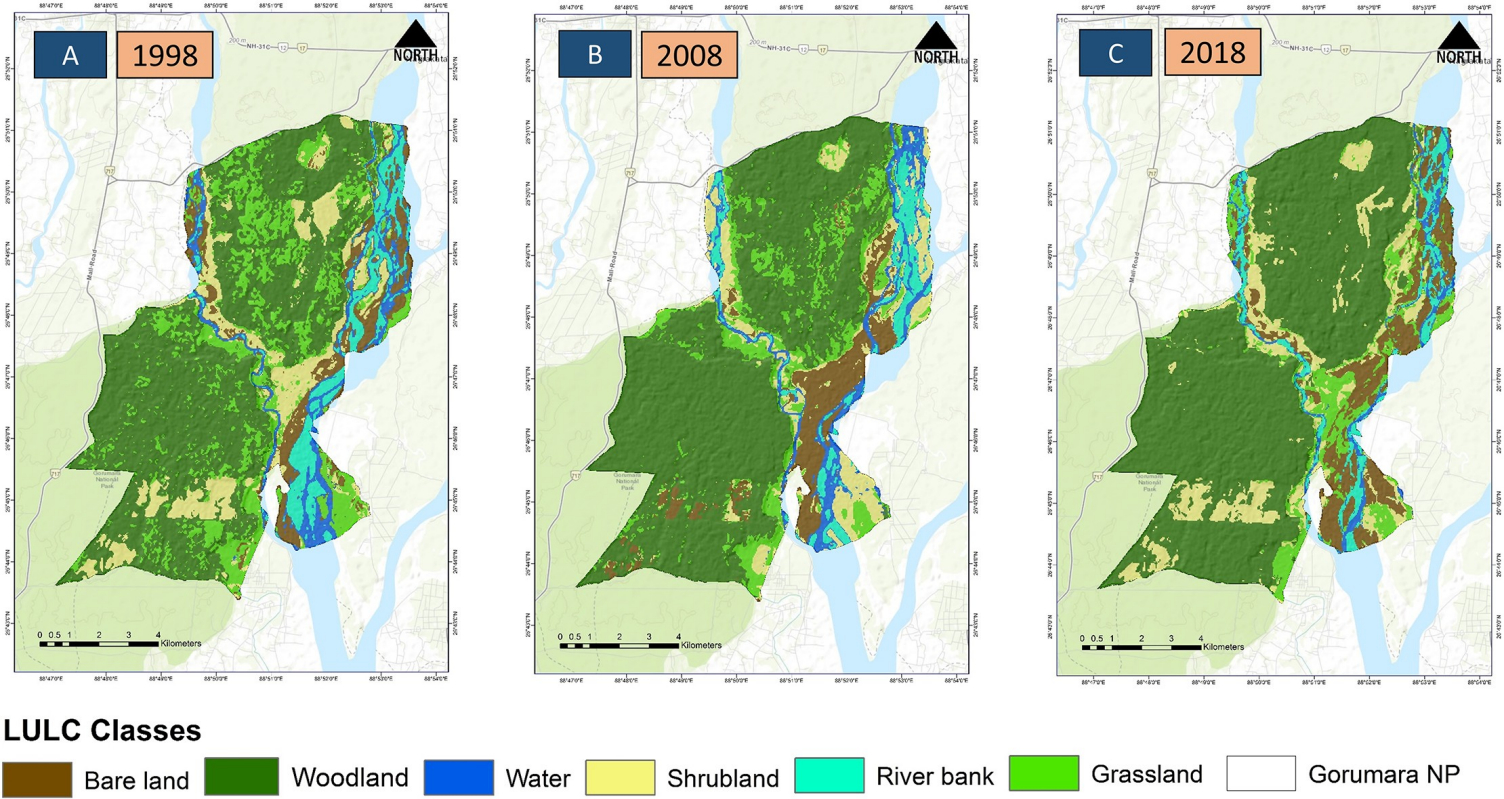
Map showing LULC across three decades in Gorumara National Park, North Bengal. **A.** LULC for 1998 **B.** LULC for 2008 **C.** LULC for 2018. The study landscape has been classified into six major land cover types (Bare ground, Grassland, Riverbank, Shrubland, Water, Woodland).

The three Landsat images were classified into six dominant LULC (Woodland, Grassland, Shrubland, Bare land, Riverbank and Water) (Fig 2). The overall accuracy for 2018 was evaluated to be 88.61% with a kappa value of 0.863 (Table A in S2 Table). The overall accuracy of the image 1998 and 2008 was 89.97% and 81.66% and kappa value was found to be 0.88 and 0.78 respectively (Table B and C in S2 Table). The comparative analysis of the three images revealed that woodland was the dominant land cover and increased in all three successive years (1998, 2008, and 2018), (Table 2), in contrast, grassland areas declined over the time. The bareland cover has increased from 6.72 km^2^ to about 9.86 km^2^ between 1998 and 2018, primarily due to loss of water bodies and narrowing of river lines.

**Table 2:** Land cover and landuse change (Δ) statistics between 1998–2008 and 2008–2018 in Gorumara National Park, North Bengal in sq. km.

LULC classes	1998	2008	2018	Δ 1998–2008	Δ 2008–2018
**Water**	5.06	5.38	2.55	0.32	-2.83
**Riverbank**	5.85	4.50	3.99	-1.35	-0.51
**Bare Land**	6.72	8.01	9.86	1.29	1.85
**Woodland**	45.58	53.26	56.44	7.68	3.18
**Grassland**	17.16	11.19	7.52	-5.96	-3.67
**Shrubland**	10.53	8.56	10.53	-1.97	1.97

The transition analysis from 1998 to 2008 indicates that the grassland cover has been converted to woodland at the most (0.517 transition value), followed by bareland to scrubland (0.375 transition value) (Table 3) (S1 Fig) (S3 Table). A similar trend was also observed from 2008 to 2018, where grassland has been converted to woodland cover (0.496 transition value). Furthermore, during 2008–2018 decade river bank got converted to bare land (0.451 transition value) and water cover has also been converted to river bank cover (0.323 transition value) (Table 4) (S1 Fig) (S3 Table).

**Table 3:** Transitional probability matrix of different LULC from 1998 to 2008.

	2008						
1998	Landcover Classes	Water	River bank	Bare ground	Woodland	Grassland	Shrubland
	Water	0.342	0.294	0.142	0.002	0.016	0.204
	River bank	0.284	0.321	0.280	0.000	0.005	0.109
	Bare ground	0.134	0.106	0.373	0.000	0.011	0.375
	Woodland	0.002	0.000	0.001	0.917	0.078	0.002
	Grassland	0.028	0.010	0.012	0.517	0.307	0.127
	Shrubland	0.048	0.021	0.277	0.245	0.208	0.201

**Table 4:** Transitional probability matrix of different LULC from 2008 to 2018.

	2018						
2008	Landcover classes	Water	River Bank	Bare Land	Woodland	Grassland	Shrubland
	Water	0.222	0.323	0.284	0.001	0.139	0.031
	Riverbank	0.157	0.326	0.451	0.000	0.058	0.008
	Bare Land	0.012	0.017	0.446	0.005	0.260	0.259
	Woodland	0.000	0.000	0.000	0.954	0.005	0.041
	Grassland	0.010	0.010	0.005	0.496	0.185	0.294
	Shrubland	0.050	0.062	0.311	0.005	0.247	0.325

### Landscape configuration change analysis

**Fig 3:**
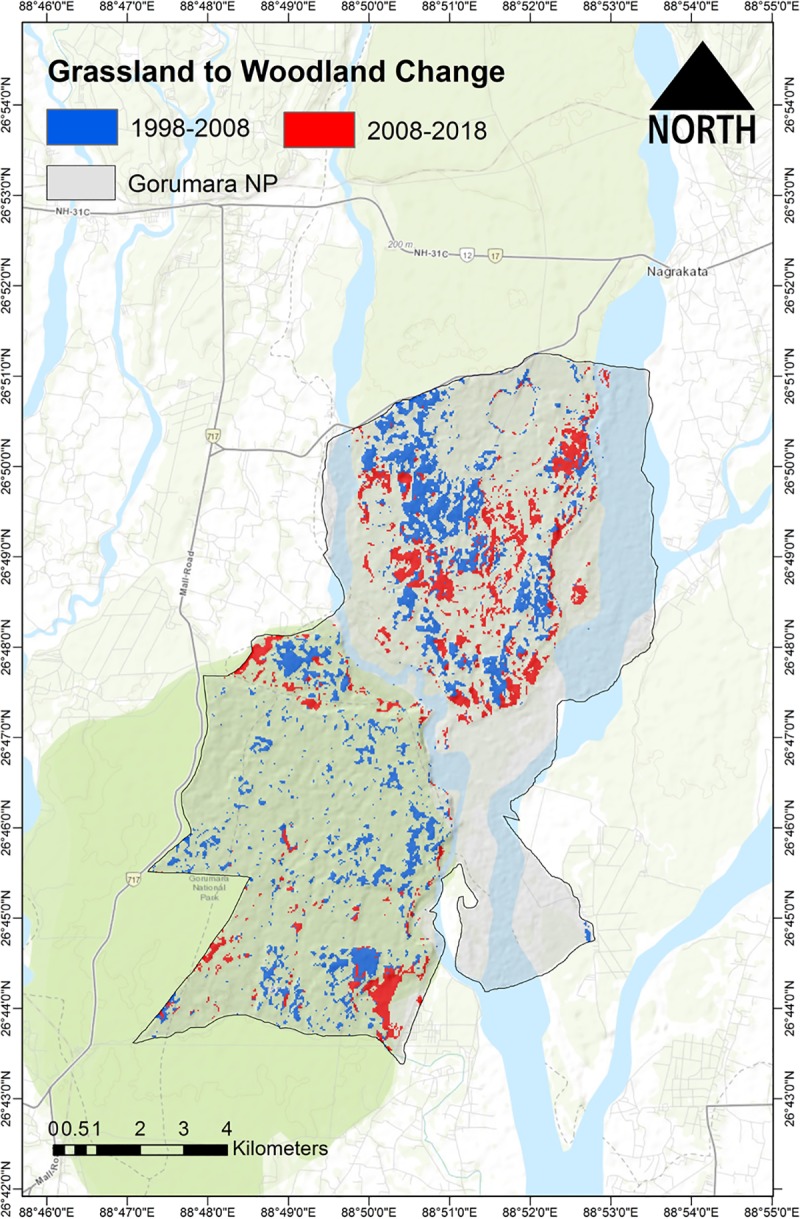
Map showing the patches of grassland converted to woodland in Gorumara National Park, North Bengal. Δ change between 1998–2008 [Red colour patches (Total area converted = 886.14 ha)] and Δ change between 2008–2018 [Blue colour patches (Total area converted = 555.57 ha)]. Topographic shading from ESRI base map (Topographic) in ArcGIS 10.6.

The change in landscape configuration indices from 1998 to 2008 and from 2008 to 2018 indicates a decrease in the number of patches (NP) and patch density (PD) (Table 5). A smaller change was observed in NP and PD from 2008 to 2018. The mean patch area (AREA MN) increased by 24% and by 2.04% from 1998 to 2008 to 2018 respectively. Both Shannon’s diversity (SHDI) and evenness (SHEI) of patches decreased. The Aggregation Index (AI) and Interspersion and Juxtaposition index (IJI) of patches increased by 5.64% and 14.67% respectively between 1998 to 2018. The Edge Density (ED) found to be following a decreasing trend starting from a reduction by 39.50% from 1998 to 2008 and by 16.26% from 2008 to 2018 suggesting a decrease in the heterogeneity of the overall landscape in multitemporal scale. Results show that about 886.14 ha of grassland have been converted into the woodland from 1998 to 2008, followed by conversion of 555.57 ha between 2008 and 2018 (Fig 3). Other major insertions have occurred between grassland to shrubland by 217.62 ha. and by 328.95 ha. followed by shrubland to bareland by 291.78 ha and by 266.4 ha. between 1998–2008 and 2008–2018, respectively (S3 Table) (S1 Fig).

**Table 5:** Summary of landscape metrics at the landscape level. **NP** (Number of patches), **ED** (Edge density), **PD** (Patch density), **AREA_MN** (Area-weighted mean patch area), **IJI** (Interspersion and juxtaposition index), **SHDI** (Shannon's diversity index), **SHEI** (Shannon's evenness index), **AI** (Aggregation index).

Metrics	1998	2008	Percentage change	Δ (1998–2008)	2018	Percentage change	Δ (2008–2018)
**NP**	1679	1276	-31.58	403	1250	-2.08	26
**PD**	18.471	14.037	-31.59	4.434	13.751	-2.08	0.286
**AREA_MN**	5.414	7.124	24.00	-1.710	7.272	2.04	-0.148
**IJI**	66.841	72.127	5.28	-5.286	81.517	9.39	-9.390
**SHDI**	1.441	1.324	-8.83	0.117	1.230	-7.58	0.093
**SHEI**	0.804	0.739	-8.83	0.065	0.687	-7.57	0.052
**AI**	85.235	89.420	4.18	-4.185	90.879	1.45	-1.459
**ED**	98.423	70.555	-39.50	27.863	60.687	-16.26	9.868

### LULC class-level configuration change analysis

The proportional area of the woodland patches in the landscape (PLAND) increased by 11.94% from 1998 to 2018 (Table 6). The number of patches (NP) along with patch density (PD) for woodland has decreased from 86 to 26 and 0.94 to 0.28 from 2008 to 2018, respectively. The Largest Patch Index (LPI- Woodland) increased by 4.91% from the years 1998 to 2018 (Table 6). Thus, the park has more woodland in a few larger patches. Area weighted Mean Shape Index (SHAPE AM-Woodland), was highest in 1998, and then fell by 81% in 2008 and to 78.23% by 2018, indicating a reduction in shape complexity of woodland patches (Table 6).

**Table 6:** Summary of landscape metrics at the class level. Representing the evaluated class level matrices for the year 1998, 2008 and 2018. Where PLAND = Percentage of landscape, NP = Number of patches, PD = Patch density, LPI = Largest patch index, AREA_AM = Area-weighted mean patch area, IJI = Interspersion and juxtaposition index, SHAPE_AM = Area-weighted mean patch shape index, CONTIG_MN = Mean Contiguity index, ENN_MN = Euclidean Nearest-Neighbour Distance.

Class	Year	1998	2008	2018	Percentage change from 1998–2008	Percentage change from 2008–2018
Woodland	PLAND	50.15	58.6	62.09	8.45	3.49
	NP	85	86	26	1.163	-230.769
	PD	0.94	0.95	0.29	1.053	-227.586
	LPI	29.68	34.55	34.59	4.87	0.04
	AREA_AM	2279.15	2718.87	2827.86	16.173	3.854
	IJI	29.26	33.1	34.15	3.84	1.05
	SHAPE_AM	12.49	6.88	3.86	-81.541	-78.238
	CONTIG_MN	0.26	0.18	0.37	-44.444	51.351
	ENN_MN	60.44	60.33	63.94	-0.182	5.646
Bare land	PLAND	7.4	8.81	10.85	1.41	2.04
	NP	284	152	158	-86.842	3.797
	PD	3.12	1.67	1.74	-86.826	4.023
	LPI	1.52	5.11	2.41	3.59	-2.7
	AREA_AM	49.73	283.23	89.19	82.442	-217.558
	IJI	82.85	81.67	81.44	-1.18	-0.23
	SHAPE_AM	2.8	3.11	2.97	9.968	-4.714
	CONTIG_MN	0.25	0.27	0.36	7.407	25.000
	ENN_MN	74.86	68.76	73.71	-8.871	6.716
Grassland	PLAND	18.87	12.31	8.28	-6.56	-4.03
	NP	717	413	389	-73.608	-6.170
	PD	7.89	4.54	4.28	-73.789	-6.075
	LPI	2.29	1.73	2.17	-0.56	0.44
	AREA_AM	67.46	49.38	70.24	-36.614	29.698
	IJI	52.24	50.75	86.24	-1.49	35.49
	SHAPE_AM	3.85	3.41	3.47	-12.903	1.729
	CONTIG_MN	0.26	0.29	0.23	10.345	-26.087
	ENN_MN	82.85	79.32	70.51	-4.450	-12.495
River bank	PLAND	6.43	4.95	4.39	-1.48	-0.56
	NP	103	101	132	-1.980	23.485
	PD	1.13	1.11	1.45	-1.802	23.448
	LPI	2.51	1.57	0.7	-0.94	-0.87
	AREA_AM	131.17	64.2	23.82	-104.315	-169.521
	IJI	36.43	31.54	62.88	-4.89	31.34
	SHAPE_AM	4.26	3.05	2.96	-39.672	-3.041
	CONTIG_MN	0.36	0.41	0.3	12.195	-36.667
	ENN_MN	71.14	82.58	77.54	13.853	-6.500
Shrubland	PLAND	11.58	9.41	11.58	-2.17	2.17
	NP	259	339	277	23.599	-22.383
	PD	2.85	3.73	3.05	23.592	-22.295
	LPI	1.8	1.97	1.51	0.17	-0.46
	AREA_AM	89.07	61.23	58.95	-45.468	-3.868
	IJI	76.81	86.96	73.15	10.15	-13.81
	SHAPE_AM	3.23	2.6	3.1	-24.231	16.129
	CONTIG_MN	0.27	0.24	0.31	-12.500	22.581
	ENN_MN	75.39	70.48	111.82	-6.967	36.970
Water	PLAND	5.57	5.92	2.81	0.35	-3.11
	NP	231	185	268	-24.865	30.970
	PD	2.54	2.04	2.95	-24.510	30.847
	LPI	1.46	1.43	0.38	-0.03	-1.05
	AREA_AM	66.75	74.49	12.31	10.391	-505.118
	IJI	73.92	77.77	75.72	3.85	-2.05
	SHAPE_AM	5.5	5.64	2.95	2.482	-91.186
	CONTIG_MN	0.18	0.2	0.18	10.000	-11.111
	ENN_MN	63.13	66.69	70.93	5.338	5.978

In the case of grassland cover, the PLAND value was highest in 1998, at 18.87%, followed by 12.31% in 2008 and 8.27% in 2018 (Table 6). NP declined from 717 patches in 1998 to 389 patches in 2018. The mean patch area (AREA MN-Grassland) declined by 36.64% in 2008 and then increased by 29.69% by 2018. The shrubland, cover has decreased from 1998 to 2018, indicated by decreases in PLAND value between 1998 and 2008 by 23.06%. However, followed by a consecutive increase again by 18.73% in the year 2018. The number of patches (NP Shrubland) has increased from 259 patches to 339 patches in 1998 to 2008 initially and then decrease significantly to 277 patches in 2018. The distribution of small and isolated patches from the complex cluster of the larger patches was measured by Euclidean nearest neighbour distance metric (ENN_AM). The most contrasting change has been observed in shrubland cover, it had increased from 75.39 in 1998 to 111.82 in 2018.

### The response of megaherbivores to landscape and class variables

**Fig 4:**
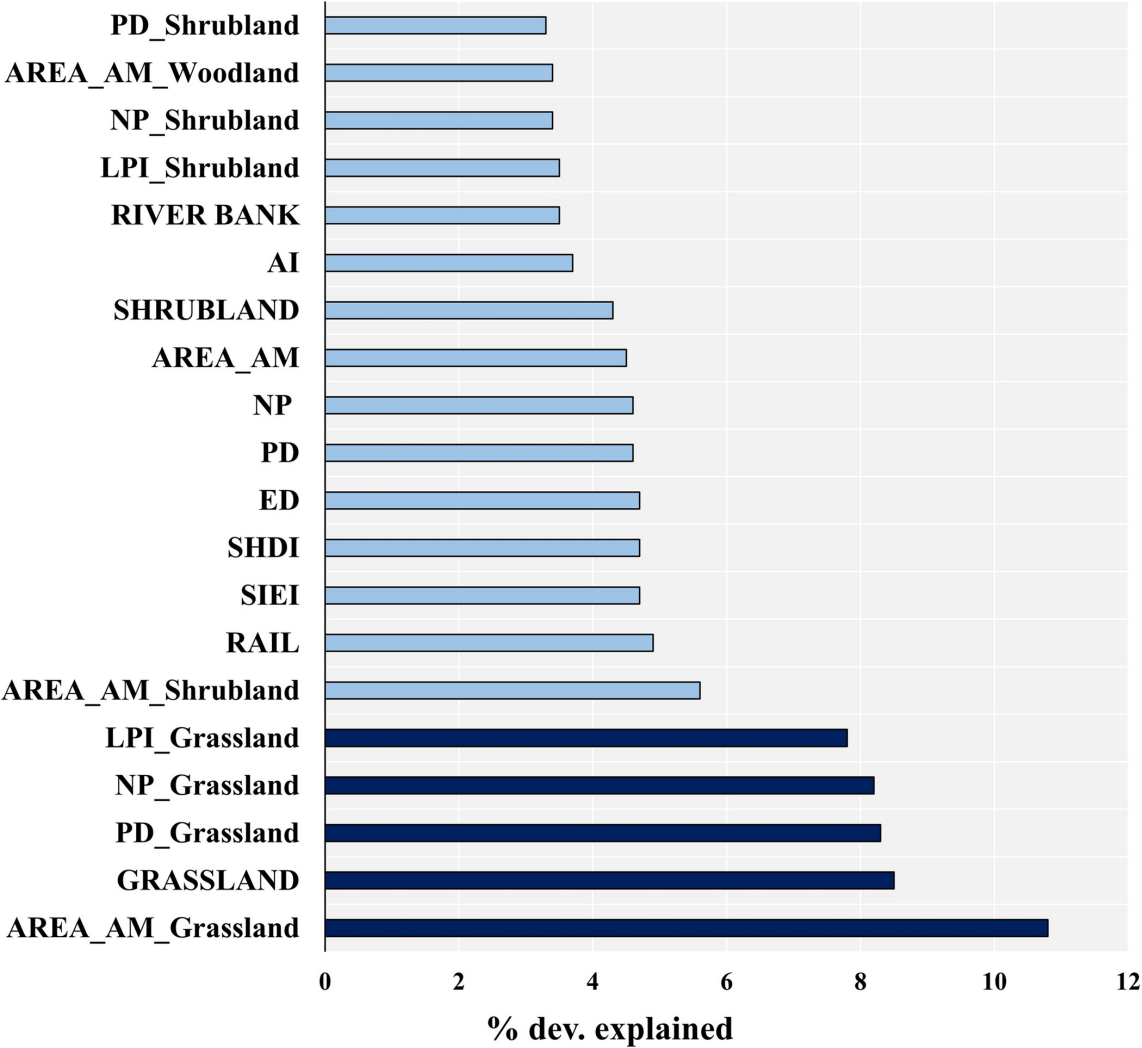
Columns display the percentage of deviation explained by top twenty covariates from Generalized Additive Modeling using the Software for Assisted Habitat Modeling (SAHM). The dark blue column represents the grassland variables which all are also the top five predictors to explain the spatial distribution of megaherbivore in GNP.

The present results indicate that Grassland, along with its class metrics are the top contributors as it explains about 43.6% deviation in the spatial distribution pattern of megaherbivores of GNP (Fig 4). Out of which Area weighted mean (AREA_AM_Grassland) for grassland contributed highest followed by the euclidian distances to grassland. Moreover, grassland, along with its metrics shows a positive response in explaining the spatial distribution of megaherbivores (S3 Fig). Further, the landscape configuration variables of grassland cover including PD, NP, LPI and Area AM responded positively to the distribution of megaherbivores in GNP (S2 Fig) (S3 Fig). The Area AM of Shrubland cover contributes about 5.6%, suggesting the importance of shrubland for the megaherbivores in GNP. Among the anthropogenic variables, rail (railway line) contributes most by 4.9% and shows a negative response with the distribution of megaherbivores (S3 Fig). The Woodland cover and it's class metrics found to contribute less in explaining the distribution of megaherbivores and the percentage of deviation explained ranged from 1.9% to 2.8%.

### Simulated future land cover for the year 2028

**Fig 5:**
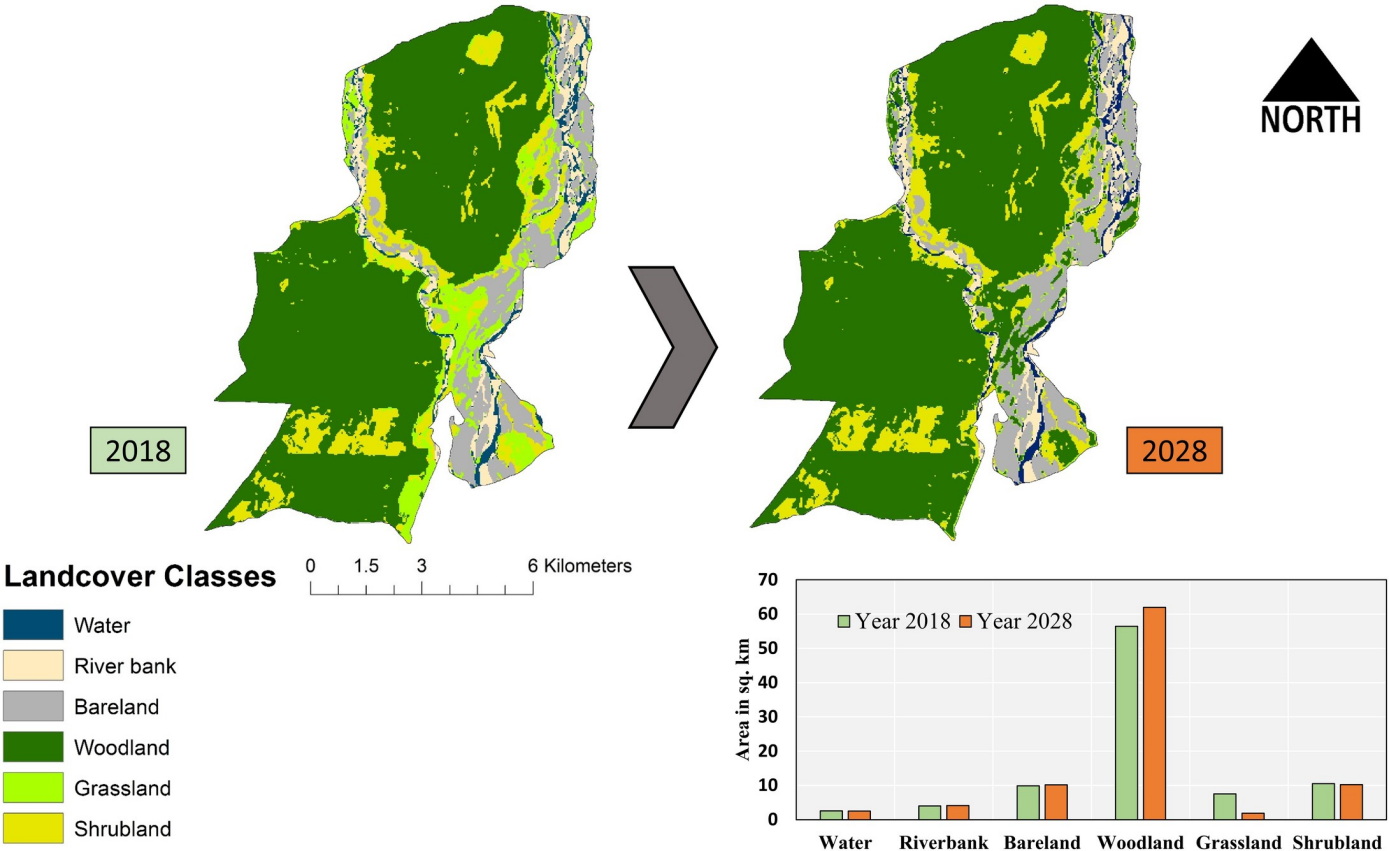
Representing the different landcover classes in GNP for the year 2018 and 2028. The upper panel left-hand image represents the landcover of 2018, and the right hand image describes the stimulated ANN (Artificial neural network) models for the year 2028. The clustered column on the right down corner represents the respective landcover classes for 2018 and 2028 in km^2^.

The future land cover for the year 2028 was simulated by adopting ANN algorithms, using the past temporal classified images of 2008 and 2018. The future land cover model was based on topographic functions. The percentage correctness and kappa (overall) was found to be 90.25% and 0.83, respectively. The comparison of model output with the classified image of 2018 predicts major change may happen in Woodland and Grassland cover types (Fig 5).

Furthermore, the model also showed a decline of about 6.23% from 2018 to 2028 in grassland. Whereas, an increment of about 6.09% has been predicted in woodland cover in 2028. Moreover due to no predicted significant change on other landcover types indicates that woodland cover may increase replacing grassland in the 2028.

## Discussions

The GNP and its surrounding landscape is one of the last habitats of rhino in India and also home to two other major herbivores, i.e. elephant and gaur which is surrounded by a network of road, railway line and expanding tourism infrastructures [84,85]. As per the latest estimates, the rhino population in the landscape has increased over the years, from eight individuals in the early 1980s to 50 as per the Forest Department latest census (S4 Table). The increasing population of rhino and other large herbivores in the small landscape of the park is posing serious conservation and management issues, such as the increase in human-animal conflicts [19].

The analysis presented here demonstrates a significant change in land cover over the three decades, with a decrease in grassland and a concomitant increase in woodland (Fig 3 and S2 Table). The decline in grassland cover is a serious conservation and management threat to the mega-herbivores, particularly rhinos, which depend upon grasslands habitats for palatable grasses and forage [86–90]. Results from the predictor inspection showed that the grassland cover along with its class metrics remained the top predictors in explaining the spatial distribution of all three mega-herbivores viz., Rhino, Elephant and Gaur in GNP (Fig 4). The significant influence of grassland on megaherbivores of GNP can be attributed to several studies, suggesting substantial consumption of grasses in the diet of megaherbivores, especially for rhinos [21–25]. Hence, loss of grassland habitats will be challenging for the park authorities in maintaining the long term viability of the population of mega-herbivores. Further, it will greatly impact the overall carrying capacity of rhino, and other mega-herbivores in the GNP will decrease, as a result of the loss of grassland habitat [91]. Furthermore, rhinos, which are primarily grazers, may also experience increased competition with other large herbivores, many of which have broader dietary preferences [92].

Results shows that shrubland cover has declined from 10.53 km^2^ from 1998 to 8.56 km^2^ in 2008, followed by a slight increase from 2008 and 2018 (Table 2). Response from predictor inspection also suggests the contribution of shrubland and its metrics in shaping the spatial distribution, as AREA_AM_shrubland was about 5.6%, followed by euclidian distance to shrubland contributes about 4.3%, and both variables showed a strong positive association with distribution of megaherbivores. Thus, it can be opined that shrubland has provided a transitional area between the most suitable grasslands and relatively less suitable woodland for megaherbivores, especially for rhinos. Therefore megaherbivores of GNP can negotiate shrubland as movement corridors, and these shrub patches may compensate for the loss of grassland in the Gorumara up to some extent [93].

Landscape-level indices found to be useful in explaining the habitat quality, the present study has identified the related contribution of landscape-level variables, i.e. SHDI and SHEI as top landscape-level contributors with the positive response, which correlates with the reducing heterogeneity in the study landscape. So the reduction in the landscape diversity by more domination of woodland will eventually lead to less suitable habitat for megaherbivores. Apart from diversity indices ED, PD NP and AI are found to be in the top twenty variables in explaining the spatial distribution of megaherbivores. Influence of such variables has been supported by studies which have highlighted the significance of landscape configuration parameters on a diverse group of animals [50,51].

The patch aggregation, as well as interspersion and juxtaposition of different classes, indicate uniformity among the patches over the three decades. The resulted increasing uniformity in the patches can be attributed to plantation activities of the West Bengal State Gorumara Wildlife Division-II in the initial working plans. Further, the increase in NP and PD of woodland cover suggests homogenous with confined spatial distribution. The reduced values of the mean shape index from 1998 to 2018 of woodland patches indicate that woodland patches have become less complex over the three decades. Moreover, other indices such as IJI, SHEI, SHDI, ED and AI at class level in the study landscape indicate that during 2008 and 2018 much of the landscape have homogenized since similar types of patches are getting aggregated with reduced shape complexity. The decrease of NP and PD of grassland patches from 1998 to 2018 is a major concern for the conservation and management of mega-herbivores in GNP. We also found that among the anthropogenic predictors, rail infrastructure contributed the most and showed a negative relation in shaping the spatial distribution of megaherbivores in GNP, this could be related with the fact that in the recent past the intensity of megaherbivores mortality because of railways network has increased considerably [94].

### Management implication for mega-herbivores

The change dynamics at both landscape and class level metrics in GNP indicate the transition of grassland to other land-use types. Which seems to be not suitable as GNP harbours a large population of sympatric megaherbivores, including elephant, rhino and gaur [25]. The prominent causes are monoculture plantations and ill-planned developmental activities, which mostly took place in the earlier working plans. Most of the conversion of natural habitat to Teak (*Tectona grandis*) and Jarul (*Lagerstroemia speciosa*) monoculture plantation took place in the areas during the VI and VII working plan before the establishment of the National Park. Such type of plantation contributes presently about 34% of the total plantation in GNP and associated forested areas [26]. The monoculture plantation of teaks does not favour the diversity of wild animals since it negatively impacts the undergrowth which is essential for the growth of fodder species [95].

Moreover, dense canopy allows very little sunlight to pass and thus inhibits the growth of light-demanding plants having browse value for ungulates. However, tree-dwellers like Giant Squirrels and certain birds may benefit from woodland habitat type, but may not support the species diversity in a broad spectrum and megaherbivores. Thus we suggest that old teak and other monoculture plantation should be kept in targeted areas on a rotation basis and canopy opening programs shall be restarted. These cleared areas should be considered for artificial regeneration of grassland to restore the carrying capacity of the ecosystem. However, the fodder plantation should not be single species oriented; instead, it should be done by using multiple species. In Jaldapara Wildlife sanctuary, a nearby protected area plantation of single grass species (*Saccharum narenga*) has impacted the overall faunal diversity composition and changing the plant community structure of the area [96]. We also recommend a systematic study on the feeding habitats of megaherbivores in GNP and surrounding landscape so that plantation preference should be given to plant species which are dominating the diet of megaherbivores of the landscape.

The relatively small area of GNP is supporting more than 900 gaurs and 50 rhino, despite a variety of anthropogenic disturbances and pressures (S3 Table). GNP has already exceeded the carrying capacity threshold for rhino, i.e. 43 individuals [19,37] and a further increase in the population will motivate animals to move out of the forested areas into the surrounding agricultural landscape in search of food [85,97]. Such movements are already causing human-wildlife conflicts in the landscape, and may antagonize the local human communities, which, in turn, may create a serious threat to the animals in question. One of the fundamental reasons for the uncontrolled increase in gaur population is food chain imbalance, in the near absence of top predators, i.e. tigers [85,97]. At present, there are no tigers left in the entire North Bengal landscape [98]. However, leopards are present in the landscape, but gaur is not their natural prey species [99]. This is leading to an ecological crisis in the landscape as rhinos are frequently found outside the park (far-flung as 50 km) to meet their foraging needs some of them staying seasonally or even permanently resulting in human-animal conflict in the landscape [15].

Furthermore, it will be imperative that tiger reintroduction in the landscape may be taken up by the forest managers for balancing the food chain. Alternatively, translocation of rhinos from GNP to other regions in the north Bengal will be a welcome step towards conservation and management of megaherbivores in the landscape. A similar initiative of rhino translocation from Pobitora Wildlife Sanctuary and Kaziranga National Park to Manas National Park through the Indian Rhino Vision 2020 (IRV 2020) program has accomplished the goal of conservation and population management of rhino [100]. We suggest establishing a rhino population in Buxa Tiger Reserve through shifting a few individuals from GNP. Apart from Buxa, the state government of West Bengal has identified a small reserve in Patlakhawa of Cooch Behar both located in the North West Bengal which can become a second home for GNP rhinos in the near future [101]. Moreover increasing connectivity by different habitat management program in the buffer forests of GNP along with increasing connectivity between Jaldapara-Gorumara and inclusion of territorial forests between Chapramari wildlife sanctuary (CWLS) and GNP in wildlife division II will be the most needed initiatives.

In addition, the forested area of GNP is crossed by an national highway (NH 31) as well as by railway lines, which is leading to loss of animals because of collisions with vehicles and trains [85,94]. These collisions of animals mostly occur on railways line between Siliguri and Alipurduar Junctions of about 150 km length. This railway line is known to traverse forest over 74 km about 44% of the length and passes through three protected areas including nine identified elephant sensitive movement corridors [94]. We recommend a reduction of train speed in this 74km stretch of the railway line to a maximum speed of 40km/hrs to avoid the collision, in conjunction with increased patrolling along the tracts jointly by railway and forest staffs for immediate implementation. Moreover, to adequately address this issue, construction of overpasses, underpasses, ecoducts and viaducts for providing safe passage at critical points and realignment of the present tract.

### Limitations and methodological considerations

For projecting simulated future landcover for the year 2028, the cellular-Automata simulation was used based on Monte Carlo algorithm. The final simulated map for transition potential was based on classified images of 2008 and 2018. The models account only the previous change and not any anthropogenic or catastrophic natural processes. Minor false classification or mixed pixel classification in some instances may impact the classification and landscape heterogeneity [102]. The present study resolution of 30m from the satellite data (Landsat TM and OLT) may be considered low for certain landcover types.

Moreover, catastrophic effects like forest fires may also contribute to the landcover changes [103–106]. For evaluating the responses of different predictor variables for megaherbivores in GNP, we did not consider the distribution of other sympatric mesoherbivores, which might influence the spatial distribution of our study species. Because of the uncertainty introduced by both the landscape modelling and predictor response segments, authors emphasize that the present study represents an approach based on the available data and possible methodical considerations for megaherbivores in the study landscape.

Further efforts could improve our findings by including range and niche overlap between sympatric megaherbivores or by incorporating other plausible biotic/abiotic variable into the model environment. Despite these assumptions and caveats, the present study represents the best available postulate to evaluate the trend and effect of landcover change at a fine resolution. The trends of past and future prediction can be used to tackle a major management problem, from which the local authorities are struggling in recent times.

## Supporting information

S1 Table Representing class-level and landscape-level metrics evaluated for landscape configuration change analysis for GNP. The study landscape has been classified into six major land cover types i.e. Bare ground, Grassland, Riverbank, Shrubland, Water and Woodland. For class metrics all land cover types have been evaluated.
(PDF)

S2 Table Accuracy assessment table for land cover classification of GNP for the year. (A) 2008, (B) 2008 and (C) 1998.
(PDF)

S3 Table Representing the transition in hectors from 1998–2008 and from 2008–2018. (PDF)

S4 Table Representing the available population and demographic data for megaherbivores of Gorumara and North Bengal. (PDF)

S1 Fig Landcover transition map of GNP. A. indicating the transition types between 1998 and 2008, B. indicating the transition types between 1998 and 2008.
(PDF)

S2 Fig **Showing the frequency distribution, respective raster visualization, spatial distribution of megaherbivores variable and response curve of** (A) Anthropogenic and topographic variables (B) Grassland variables, (C) Woodland variables, (D) Shrubland variables and (E) Landscape level variables.
(PDF)

S3 Fig Bar graph representing percentage of deviation explained by all covariates from Generalized Additive Modeling using the Software for Assisted Habitat Modeling (SAHM). (PDF)

S4 Fig Methodological flow chart of the study. (PDF)

## References

[1] Roy PS, Tomar S. Landscape cover dynamics in Meghalaya. Int J Remote Sens. 2001; 22: 3813–3825. 10.1080/01431160010014008

[2] Nagendra H, Munroe DK, Southworth J. From pattern to process: landscape fragmentation and the analysis of land use/land cover change. Agric Ecosyst Environ. 2004; 101: 111–115.

[3] Zheng D, Wallin DO, Hao Z. Rates and patterns of landscape change between 1972 and 1988 in the Changbai Mountain area of China and North Korea. Landscape Ecol. 1997; 12: 241–254.

[4] Srivastava S, Singh TP, Singh H, Kushwaha SPS, Roy PS. Assessment of large-scale deforestation in Sonitpur district of Assam. Curr Sci. 2002; 82: 1479–1484.

[5] Ostrom E, Nagendran H. Insights on linking forests, trees, and people from the air, on the ground and in the laboratory. Proc. Natl. Acad. Sci. U. S. A. 2006; 103: 224–231. 10.1073/pnas.0506736102 17088538 PMC1838564

[6] Sarma PK, Talukdar BK, Sarma K, Barua M. Assessment of habitat change and threats to the greater one-horned rhino (*Rhinoceros unicornis*) in Pabitora Wildlife Sanctuary, Assam, using multi-temporal satellite data. Pachyderm. 2009; 46: 18–24.

[7] Kachhwaha TS. Temporal and multi-sensor approach in forest vegetation mapping and corridor identification for effective management of Rajaji National Park, Uttar Pradesh, India. Int J Remote Sens. 1993; 14; 3105–3114.

[8] Nagendra H, Tucker C, Carlson L, Southworth J, Karmacharya M, Karna B. Monitoring parks through remote sensing: Studies in Nepal and Honduras. Environ Manage. 2004; 34: 748–760. 10.1007/s00267-004-0028-7 15633028

[9] Sarma PK, Lahkar BP, Ghosh S, Rabha A, Das JP, Nath NK, et.al Land-use and land-cover change and future implication analysis in Manas National Park, India using multi-temporal satellite data. Curr Sci. 2008; 95:223–227.

[10] Bayarsaikhan U, Boldgiv B, Kim KR, Park KA, Lee D. Change detection and classification of land cover at Hustai National Park in Mongolia. Int J Appl Earth Obs Geoinf. 2009; 11: 273–280.

[11] Kushwaha SPS, Roy PS, Azeem A, Boruah P, Lahan P. Land area changes and rhino habitat suitability analysis in Kaziranga NP, Assam. Tiger Paper. 2000; 27: 9–16.

[12] Sukumar R. The Asian elephant: ecology and management. 1st ed Cambridge: Cambridge University Press; 1989.

[13] Dinerstein E. An ecological survey of the Royal Karnali-Bardia Wildlife Reserve, Nepal. Part I: vegetation, modifying factors, and successional relationships. Biol. Conserv. 1979; 15: 127–150.

[14] Fritz H, Duncan P, Gordon IJ, Illius AW. Megaherbivores influence trophic guilds structure in African ungulate communities. Oecologia. 2002; 131: 620–625. 10.1007/s00442-002-0919-3 28547558

[15] Mukherjee N. A Brief Appraisal of Human wildlife conflict in Jalpaiguri and Alipurduar districts of West Bengal. Int J Sci Res Pub. 2016; 6: 131–136.

[16] Liu Z, He C, Wu J. The relationship between habitat loss and fragmentation during urbanization: an empirical evaluation from 16 world cities. PLoS ONE 11(4): e0154613 10.1371/journal.pone.0154613 27124180 PMC4849762

[17] Haddad NM, Brudvig LA, Clobert J, Davies KF, Gonzalez A, Holt RD, et al Habitat fragmentation and its lasting impact on Earth’s ecosystems. Sci. Adv. 2015; 1: e1500052 10.1126/sciadv.1500052 . 26601154 PMC4643828

[18] Wildlife Wing: Directorate of Forests Govt. of West Bengal. [cited 12.02.2019] Available from: https://www.wildbengal.com/.

[19] Mallick JK. Ecological crisis vis-à-vis intraspecific conflict: a case study with rhinos in Jaldapara and Gorumara National Parks, West Bengal, India. In: Gupta VK, Verma AK, editors. Animal Diversity, Natural History and Conservation; 2015 pp. 335–366.

[20] Thapa K, Williams AC. Khaling SB. Observations on habitat preferences of translocated rhinos in Bardia National Park and Suklaphanta wildlife reserve, Nepal. Pachyderm. 2009; 45:108–114.

[21] Laurie WA. The ecology and behaviour of the greater one-horned rhinoceros. PhD. Dissertation, Cambridge University. 1978.

[22] Fjellstad JI and Steinheim G. Diet and habitat use of greater Indian one-horned rhinoceros (*Rhinoceros unicornis*) and Asian elephants (*Elephas maximus*) during dry season in Babai Valley, Royal Bardia National Park, Nepal. M.Sc. Thesis, Agricultural University of Norway. 1996.

[23] Hazarika BC, Saikia PK. Food Habit and Feeding Patterns of Great Indian One-Horned Rhinoceros (*Rhinoceros unicornis*) in Rajiv Gandhi Orang National Park, Assam, India. Int Sch Res Notices: Zool. 2012; 1–11. 10.5402/2012/259695

[24] Konwar P, Saikia MK, Saikia PK. Abundance of food plant species and food habits of *Rhinoceros unicornis* Linn. In Pobitora Wildlife Sanctuary, Assam, India. J Threat Taxa. 2009; 1: 457–460.

[25] Ghosh SB. Biodiversity and wild fodder of Gorumara National Park in West Bengal, India. J. Ecol Env. 2012; 3: 18–35.

[26] Debnath HS, Alfred JRB, Chowdhury BR, Saha R, Maity KLM, Mukherjee A. Impact of habitat management practices, especially canopy manipulation and grassland restoration, on the habitat use pattern of herbivores and the herbivores-carrying capacity in Jaldapara NP, Gorumara NP and Mahananda WLS. 2017 [cited 05 May 2019]. In Inception report from Nature Environment & Wildlife Society (NEWS). Available from: https://www.wbfdpj.org/project/upload/inception6.pdf. 10.1586/17434440.4.6.815

[27] Peden DG, Van Dyne GM, Rice RW, Hansen RM. The trophic ecology of Bison on short grass plains. Nat Res Ecol. 1974; 11: 489–497.

[28] Nayak BK, Patra AK. Food and feeding habits of Indian Bison, (Smith, 1827) in Kuldiha Wildlife Sanctuary, Balasore, Odisha, India and its conservation. Int Res J Biol Sci. 2015; 4: 73–79.

[29] Chetri M. Food habit, habitat utilization and conservation of Gaur (*Bos gaurus*) in Parsa Wildlife Reserve Nepal. M.Sc. Thesis, Department of Zoology Tribhuvan University Kathmandu Nepal. 1999.

[30] Chetri M. Diet Analysis of Gaur (*Bos gaurus* Smith, 1827) by Micro Histological Analysis of Fecal Samples in Parsa Wildlife Reserve. Our Nat. 2007; 4: 20–28.

[31] Haleem A, Ilyas O. Food and Feeding Habits of Gaur (*Bos gaurus*) in Highlands of Central India: A Case Study at Pench Tiger Reserve, Madhya Pradesh (India). Zool Sci. 2018; 35: 57–67. 10.2108/zs170097 29417898

[32] McKay GM. Behaviour and ecology of the Asiatic elephant in South-eastern Ceylon. Smithson. Contrib. Zool. 1973; 125: 1–113.

[33] Sivaganesan N, Johnsingh AJT. Food resources crucial to the wild elephants in Mudumalai Wildlife Sanctuary, Tamil Nadu, India In: Daniel JC, Datye HS, editors. A Week with Elephants. Proceedings of the International Seminar on the Conservation of Asian Elephant. Bombay Natural History Society, Oxford University Press, Bombay, India; 1995 pp. 405–423.

[34] Baskaran N, Balasubrmanian M, Swaminathan S, Desai AA. Feeding ecology of the Asian elephant (*Elephas maximus* Linnaeus) in the Nilgiri biosphere reserve, southern India. J. Bombay Nat. Hist. Soc. 2010; 107: 3–13.

[35] Roy M, Chowdhury S. Foraging Ecology of the Asian Elephant in Northern West Bengal. Gajah. 2014; 40: 18–25.

[36] Molur S, Sukumar R, Seal U, Walker S. Report: Population and Habitat Viability Assessment (P.H.V.A) Workshop: Great Indian One-horned Rhinoceros: Jaldapara. CBSG, Coimbatore; 1995. 85 pp.

[37] Martin EB. Policies that work for rhino conservation in West Bengal. Pachyderm. 2006; 41: 74–84.

[38] Bierwagen BG. Connectivity in urbanizing landscapes: The importance of habitat configuration, urban area size, and dispersal. Urban Ecosyst. 2007; 10: 29–42. 10.1007/s11252-006-0011-6

[39] Turner MG, Gardner RH. Quantitative techniques in landscape ecology-the analysis and interpretation of landscape heterogeneity. 1st ed New York: Springer-Verlag press; 1991.

[40] Worboys GL. Concept, purpose and challenges In: Worboys GL, Lockwood M, Kothari A, Feary S, Pulsford I, editors. Protected Area Governance and Management. Canberra: ANU Press; 2015 pp. 9–42.

[41] Heino M, Kummu M, Makkonen M, Mulligan M, Verburg PH, Jalava M, et al Forest loss in protected areas and intact forest landscapes: a global analysis. PLoS ONE. 2015: 10(10): e0138918 10.1371/journal.pone.0138918 26466348 PMC4605629

[42] Barnes BV, Zak DR, Denton SR, Spurr SH. Forest ecology. 4th ed New York: John Wiley & Sons Inc; 1998.

[43] Turner MG, Gardner RH, O’Neil RV. Ecological dynamics at broad scales; ecosystems and landscapes. BioScience Supplement. 1995; 45:S-29–S-33. 10.2307/1312440

[44] Goodland RJA, Daly HE, Serafy S. The urgent need for rapid transition to global environmental sustainability. Environ. Conserv. 1993; 20: 297–309. 10.1017/S0376892900023481

[45] Haila Y. A conceptual genealogy of fragmentation research: from island biogeography to landscape ecology. Ecol. Appl. 2002; 12: 321–334. 10.1890/1051-0761(2002)012[0321:ACGOFR]2.0.CO;2

[46] Kupfer JA, Malanson GP, Franklin SB. Not seeing the ocean for the islands: the mediating influence of matrix-based processes on forest fragmentation effects. Glob. Ecol. Biogeogr. 2006; 15: 8–20. 10.1111/j.1466-822X.2006.00204.x

[47] Laurance WF. Theory meets reality: how habitat fragmentation research has transcended island biogeographic theory. Biol Cons. 2008; 141: 1731–1744. 10.1016/j.biocon.2008.05.011

[48] Chen J, Deng X, Zhang L, Bai Z. Diet composition and foraging ecology of Asian elephants in Shangyong, Xishuangbanna, China. Acta Ecologica Sinica. 2006; 26: 309–316. 10.1016/S1872-2032(06)60006-1

[49] Sitompul AF, Griffin CR, Rayl ND, Fuller TK. Spatial and temporal habitat use of an Asian Elephant in Sumatra. Animals. 2013; 3: 670–679. 10.3390/ani3030670 26479527 PMC4494438

[50] Wadey J, Beyer HL, Saaban S, Othman N, Leimgruber P, Campos-Arceiz A. Why did the elephant cross the road? The complex response of wild elephants to a major road in Peninsular Malaysia. Biol Cons. 2018; 218: 91–98. 10.1016/j.biocon.2017.11.036

[51] Huang C, Li X, Khanal L, Jiang X. Habitat suitability and connectivity inform a co-management policy of protected area network for Asian elephants in China. PeerJ. 2019; 19: 7:e6791. 10.7717/peerj.6791 31041155 PMC6476284

[52] Verburg PH, Eck JRV, Hijs TCD, Dijst MJ, Schot P. Determination of land use change patterns in the Netherlands. Environ Plann B. 2004; 31: 125–150.

[53] Agrawal C, Green G, Grove J, Evans T, Schweik C. A review and assessment of land-use change models: dynamics of space, time, and human choice. Delaware OH: USDA Forest Service 2002; 10.2737/NE-GTR-297

[54] Sinha P, Kimar L. Markov land cover change modeling using pairs of time-series satellite images. Photogrammetric Engineering & Remote Sensing, 2013; 79: 1037–1051.

[55] Weng Q. Land use change analysis in the Zhujiang Delta of China using satellite remote sensing, GIS and stochastic modelling. J Environ Manage. 2002; 64: 273–284. 10.1006/jema.2001.0509 12040960

[56] Calabrese A, Calabrese JM, Songer M, Wegmann M, Hedges S, Rose R, et al Conservation status of Asian elephants: the influence of habitat and governance. Biodivers. Conserv. 2017; 26: 2067–2081. 10.1007/s10531-017-1345-5

[57] Weiers S, Bock M, Wissen M, Rossner G. Mapping and indicator approaches for the assessment of habitats at different scales using remote sensing and GIS methods. Landscape Urban Plann. 2004; 67: 43–65.

[58] Weng YC. Spatiotemporal changes of landscape pattern in response to urbanization. ‎ Landscape Urban Plann. 2007; 81: 341–353.

[59] Rodgers WA, Panwar HS, Mathur VB. Wildlife Protected Areas in India: A Review (Executive Summary). 1st ed Dehradun: Wildlife Institute of India; 2002.

[60] Sanyal AK, De JK, Das RP, Venkataraman K. Feasibility study regarding re-introduction of pygmy hog (Porcula salvania Hodgson, 1847) at Gorumara National Park, Jalpaigui, West Bengal. Rec Zool Surv India. 2013; 113: 1–24.

[61] Tapas D, Bimal D. Management Plan of Gorumara National Park. West Bengal: Wild Life Circle, Government of West Bengal; 2017.

[62] Champion HG, Seth SK. A revised survey of forest types of India. 1st ed Delhi: Manager of Publications; 1968.

[63] Ghatak S, Bhutia PT, Mitra A, Raha AK. Time Series Study of Rhino Habitat and its Impact on Rhino Population in Gorumara National Park through Remote Sensing Technology. Int. J environ agric biotechnol. 2016; 1: 328–333. 10.22161/ijeab/1.3.3

[64] Congedo L. Semi-Automatic Classification Plugin Documentation; 2016. Release 6.0.1.1. Available from: 10.13140/RG.2.2.29474.02242/1 Cited 17 May 2019.

[65] Nguyen TA, Le PMT, Pham TM, Hoang HTT, Nguyen MQ, Ta HQ, et al Toward a sustainable city of tomorrow: a hybrid Markov–Cellular Automata modeling for urban landscape evolution in the Hanoi city (Vietnam) during 1990–2030. Environ Dev Sustain. 2019; 21: 429–446. https://doi.org/10. 1089/sus.2017.29092.aml

[66] Liping C, Yujun S, Saeed S. Monitoring and predicting land use and land cover changes using remote sensing and GIS techniques—A case study of a hilly area, Jiangle, China. 2018; PLoS ONE 13(7): e0200493 10.1371/journal.pone.0200493 30005084 PMC6044539

[67] Saputra, & Lee. (2019). Prediction of Land Use and Land Cover Changes for North Sumatra, Indonesia, Using an Artificial-Neural-Network-Based Cellular Automaton. Sustainability. 2019; 11: (11), 3024 10.3390/su11113024

[68] Rahman MTU, Tabassum F, Rasheduzzaman M, Saba H, Sarkar L, Ferdous J, et.al Temporal dynamics of land use/land cover change and its prediction using CA-ANN model for southwestern coastal Bangladesh. Environ Monit Assess. 2017; 189: 3–18. 10.1007/s10661-017-6272-0 29039035

[69] Mtui DT, Lepczyk CA, Chen Q, Miura T, Cox LJ. Assessing multi-decadal land-cover- land-use change in two wildlife protected areas in Tanzania using Landsat imagery. PLoS ONE. 2017; 12(9): e0185468 10.1371/journal.pone.0185468 28957397 PMC5619789

[70] McGarigal K, Cushman SA, Neel MC, Ene E. FRAGSTATS: Spatial Pattern Analysis Program for Categorical Maps. Computer Software Program, University of Massachusetts, Amherst, Mass, USA. 2002. Available from: http://www.umass.edu/landeco/research/fragstats/fragstats.html.

[71] Haines-Young R., Chopping M. Quantifying landscape structure: a review of landscape indices and their application to forested landscapes. Prog Phys Geogr. 1996; 20: 418–445.

[72] Dale VH, Pearson SM. Quantifying habitat fragmentation due to land-use change in Amazonia In Laurance WF, Bierregaard RO, editors. Tropical Forest Remnants: ecology, management, and conservation of fragmented communities Chicago: Chicago Press; 1997.

[73] Hastie TJ, Tibshirani RJ. Generalized additive models. 1st ed London: Chapman and Hall; 1990 10.1002/sim.4780110717

[74] Morisette JT, Jarnevich CS, Holcombe TR, Talbert CB, Ignizio D, Talbert MK et al VisTrails SAHM: Visualization and workflow management for species habitat modeling. Ecography. 2013; 36: 129–135. 10.1111/j.1600-0587.2012.07815.x

[75] Tao M, Xie P, Chen J, Qin B, Zhang D, Niu Y, et al Use of a generalized additive model to investigate key abiotic factors affecting microcystin cellular quotas in heavy bloom areas of lake Taihu. PLoS ONE. 2012; 7(2): e32020 10.1371/journal.pone.0032020 22384128 PMC3285656

[76] Jogun T. The simulation model of land cover change in the Požega-Slavonia County. Diploma thesis, Faculty of Science, Department of Geography. 2016. Available from: http://digre.pmf.unizg.hr/4908/

[77] Li T, Li W. Multiple land use change simulation with Monte Carlo approach and CA-ANN model, a case study in Shenzhen, China. Environ Syst Res. 2015; 4:1 10.1186/s40068-014-0026-6.

[78] NEXTGIS. MOLUSCE—quick and convenient analysis of land cover changes; 2013. E-Print. Available from: https://nextgis.com/blog/molusce. Accessed 10 July 2019.

[79] Gessler PE, Moore ID, McKenzie NJ, Ryan PJ. Soil-landscape modeling and spatial prediction of soil attributes. Int J Geogr Inf Sci. 1995; 9: 421–432. 10.1080/02693799508902047

[80] Moore ID, Lewis A, Gallant JC. Terrain attributes: estimation methods and scale effectsaffected: Jakeman AJ Beck MB, MCaleer MJ editors. Modelling Change in Environmental Systems. Chichester: Wiley; 1993 pp. 189–214.

[81] McCune B, Keon D. Equations for potential annual direct incident radiation and heat load index. J Veg Sci. 2002; 13: 603–606. 10.1111/j.1654-1103.2002.tb02087.x

[82] Iverson LR, Dale ME, Scott CT, Prasad A. A GIS-derived integrated moisture index to predict forest composition and productivity of Ohio forests (U.S.A.). Landsc Ecol. 1997; 12: 331–348.

[83] Evans JS, Oakleaf J, Cushman SA, Theobald D. An ArcGIS Toolbox for Surface Gradient and Geomorphometric Modeling, version 2.0–0; 2014. E-print. Available from: http://evansmurphy.wix.com/evansspatial. Cited 10 July 2019.

[84] Roy M. A Spatial and Temporal Analysis of Elephant-Human Conflict at Gorumara and Jalpaiguri Forest Divisions of Northern West Bengal. J Wildl Res. 2017; 5: 41–49.

[85] Chakraborty S. Human-Animal Conflicts in Northern West Bengal: Losses on both sides. Int J Pure App Biosci 2015; 3: 35–44.

[86] Prater SH. The book of Indian animals. 3rd ed Bombay: Bombay natural history society; 1971.

[87] Kafley H, Khadka M, Sharma M. Habitat evaluation and suitability modeling of Rhinoceros unicornis in Chitwan National Park, Nepal: A geospatial approach. A report submitted to Aloca Foundation, Institute of International Education, World Wildlife Fund, USA; 2008.

[88] Jnawali SR. Population ecology of greater one-horned Rhinoceros (*Rhinoceros unicornis*) with particular emphasis on habitat preference, food ecology and ranging behavior of a reintroduced population in Royal Bardiya National Park in Low land Nepal. PhD. Thesis, Agricultural University of Norway. 1995. Available from:http://www.rhinoresourcecenter.com/index.php?s=1&act=refs&CODE=ref_ detail&id=1211098973.

[89] Kandel RC. Aspects of foraging activity, habitat use and demography of rhinoceros (*Rhinoceros unicornis* Linn) in Royal Chitwan National Park, Nepal. M.Sc. dissertations, Wildlife Institute of India, Dehradun. 2003.

[90] Kandel RC, Jhala YV. Demographic Structure, Activity patterns, Habitat Use and Food Habits of Rhinoceros unicornis in Chitwan National Park, Nepal. J Bombay Nat Hist Soc. 2008; 105: 5–13.

[91] Sarma PK, Mipun BS, Talukdar BK, Singh H, Basumatary AK, Das AK. et.al Assessment of habitat utilization pattern of rhinos (*Rhinoceros unicornis*) in Orang National Park, Assam, India. Pachyderm. 2012; 51: 38–44.

[92] Jarman PJ, Sinclair ARE. Feeding strategy and pattern of resource-partitioning in ungulates In: Sinclair ARE, Norton-Griffiths M, editors. Serengeti: dynamics of an ecosystem. Chicago: University of Chicago; 1979 pp. 130–163.

[93] Rimal S, Adhikari H, Tripathi S. Habitat suitability and threat analysis of Greater One-horned Rhinoceros (Rhinoceros unicornis Linnaeus, 1758) (Mammalia: Perissodactyla: Rhinocerotidae) in Rautahat District, Nepal. J Threat Taxa. 2018; 10: 11999–12007. 10.11609/jott.3948.10.8.11999-12007

[94] Roy M, Sukumar R. Railways and Wildlife: A Case Study of Train-Elephant Collisions in Northern West Bengal, India In: Borda-de-Água L, Barrientos R, Beja P, Pereira HM, editors. Railway Ecology. Cham: Springer Nature; 2017 pp. 157–177. 10.1007/978-3-319-57496-7_10

[95] Bremer LL, Farley KA. Does plantation forestry restore biodiversity or create green deserts? A synthesis of the effects of land-use transitions on plant species richness. Biodivers Conserv. 2010; 19: 3893–3915. 10.1007/s10531-010-9936-4

[96] Ghosh C, Das AP. Rhino-Fodders in Jaldapara Wildlife Sanctuary in Duars of West Bengal, India. Our Nature. 2007; 5: 14–20. 10.3126/on.v5i1.792

[97] Manoj K, Bhattacharyya R, Padhy PK. Forest and Wildlife Scenarios of Northern West Bengal, India: A Review. Int. Res. J. Biological Sci. 2013; 2: 70–79.

[98] Jhala YV, Qureshi Q, Naya AK. Status of tigers, co-predators and prey in India 2018. Summary Report. National Tiger Conservation Authority, Government of India, New Delhi & Wildlife Institute of India, Dehradun; 2019. TR No./2019/05. Available from: https://projecttiger.nic.in/WriteReadData/PublicationFile/Tiger% 20Status%20Report_XPS220719032%20%20new%20layout(1).pdf. Cited 10 July 2019

[99] Bhattarai BP, Kindlmann P. Interactions between Bengal tiger (*Panthera tigris*) and leopard (*Panthera pardus*): implications for their conservation. Biodivers Conserv. 2012; 21: 2075–2094. 10.1007/s10531-012-0298-y

[100] Agarwalla RP. IRV 2020 overview In: Ellis S, Miller PS, Agarwalla RP, Yadava MK, Ghosh S, Sivakumar P. et.al (Eds.) Indian Rhino Vision 2020 Population Modeling Workshop Final Report. Guwahati, Assam, India International Rhino Foundation; 2015 pp. 4–72.

[101] Dasgupta S. Rhinos to be relocated to Two New Sites in West Bengal. The WIRE. 23 11 2016 [Cited 2019 March 19] Available from: https://thewire.in/politics/rhinos-relocation-jaldapara-gorumara-buxa

[102] Verbyla DL, Boles SH. Bias in land cover change estimates due to misregistration. Int J. Remote Sens. 2000; 21: 3553–3560. 10.1080/014311600750037570

[103] Sankaran M, Ratnam J, Hanan N. Woody cover in African savannas: the role of resources, fire and herbivory. Glob. Ecol. Biogeogr. 2008; 17: 236–245. 10.1111/j.1466-8238.2007.00360.x

[104] Scanlon TM, Albertson JD, Caylor KK, Williams CA. Determining land surface fractional cover from NDVI and rainfall time series for a savanna ecosystem. Remote Sens. Environ. 2002; 82: 376–388. 10.1016/S0034-4257(02)00054-8

[105] Kikula IS. The influence of fire on the composition of miombo woodland of sw Tanzania. Oikos. 1986; 46: 317–324. 10.2307/3565829

[106] Holdo RM. Elephant herbivory, frost damage and topkill in Kalahari sand woodland savanna trees. J. Veg. Sci. 2006; 17: 509–518. 10.1111/j.1654-1103.2006.tb02472.x

