# Supplementary material for: Changing landscape configuration demands ecological planning: Retrospect and prospect for megaherbivores of North Bengal
Source: PLoS One. 2019 Dec 19;14(12):e0225398. doi: 10.1371/journal.pone.0225398 (PMC6922392; doi:10.1371/journal.pone.0225398)
Supplement: S1 Table — The study landscape has been classified into six major land cover types i.e. Bare ground, Grassland, Riverbank, Shrubland, Water and Woodland. For class metrics all land cover types have been evaluated. (PDF) [file pone.0225398.s001.pdf]

S1 Table. Representing class-level and landscape-level metrics evaluated for landscape configuration change analysis for GNP. The study landscape has been classified into six major land cover types i.e. Bare ground, Grassland, Riverbank, Shrubland, Water and Woodland. For class metrics all land cover type have been evaluated.

**Class metrics**			
	**DESCRIPTION**	**Aspect**	**UNIT**
**PLAND** (Percentage of landscape)	Proportion of the landscape occupied by a given patch type. PLAND have been calculated for all the landcover types.	Habitat fragmentation and habitat loss	Percent
**NP** (Number of patches)	Total number of patches in the landscape for a given patch type. PD have been calculated for all the landcover types.	Fragmentation	Information value (No Unit)
**PD** (Patch density)	The number of patches of the given patch type divided by total landscape area PD have been calculated for all the landcover types.	Fragmentation	Number per 100 hectares
**LPI** (Largest patch index)	Percentage of total landscape area occupied by the largest patch for a given patch type. LPI have been calculated for all the landcover types.	Dominance	Percent
**AREA_AM** (Area-weighted mean patch area)	AM (area-weighted mean area) equals the weighted average of patches of the given type, when patch contributions to the average are weighted by patch area. AREA_AM have been calculated for all the landcover types.	Class proportions	Information value (No Unit)
**IJI** (Interspersion and juxtaposition index)	Observed interspersion of a given patch type, divided by the maximum possible interspersion for the given number of LULC classes. IJI is based on patch adjacencies. IJI have been calculated for all the landcover types.	Uniformity in class level configuration	Percent
**SHAPE_AM** (Area-weighted mean patch shape index)	Average shape index of patches of a given patch type, weighted by overall area for the patch type. SHAPE_AM have been calculated for all the landcover types.	Shape complexity	Information value (No Unit)
**CONTIG_MN** (Mean Contiguity index)	Measure of contiguity based on patch size and connectivity to like patches. CONTIG_MN have been calculated for all the landcover types.	Spatial connectedness	Information value (No Unit)

**(ENN_AM)** (Euclidean Nearest-Neighbour Distance)	Distance to the nearest neighbouring patch of the same type, based on cell center to cell center.	Patch isolation	Meters
**Landscape-level metrics**			
	**DESCRIPTION**	**INDICATOR OF**	**UNIT**
**NP** (Number of patches)	Total number of patches in the landscape	Fragmentation	Information value (No Unit)
**ED** (Edge density)	Sum of lengths of all edge segments involving the given landscape, divided by total landscape area.	Heterogeneity in the landscape	Meters per hectare
**PD** (Patch density)	The number of patches in the landscape divided by total landscape area.	Fragmentation	Number per 100 hectares
**AREA_MN** (Area-weighted mean patch area)	Measures central tendency for patch area across the entire landscape by giving equal weightage regardless of patch size.	Landscape proportions	Information value (No Unit)
**IJI** (Interspersion and juxtaposition index)	Observed interspersion over the maximum possible interspersion for the given number of patch types at landscape level	Uniformity in landscape configuration	Percent
**SHDI** (Shannon's diversity index)	Proportional abundance of every patch of the landscape multiplied by that proportion.	Landscape Diversity	Information value (No Unit)
**SHEI** (Shannon's evenness index)	Measures the dominance of patches within the total area.	Landscape Evenness	Information value (No Unit)
**AI** (Aggregation index)	Calculates the like adjacencies of different pairs of patch types involving the focal class	Aggregation	Per cent
