# Supplementary material for: Changing landscape configuration demands ecological planning: Retrospect and prospect for megaherbivores of North Bengal
Source: PLoS One. 2019 Dec 19;14(12):e0225398. doi: 10.1371/journal.pone.0225398 (PMC6922392; doi:10.1371/journal.pone.0225398)
Supplement: S2 Table — (A) 2008, (B) 2008 and (C) 1998. (PDF) [file pone.0225398.s002.pdf]

S2 Table. Accuracy assessment table for land cover classification of GNP for the year (A) 2008, (B) 2008 and (C) 1998.

(A) Year 2018.

Land cover classification for GNP									
Contingency Matrix									
		Observed land cover Classes						Grand Total:	User's Accuracy:
		Water	Bare land	River bank	Grassl and	Shrubland	Woodland		
Mapped land cover	Water	55	0	6	0	0	0	61	90.163
	Bare land	1	57	3	3	0	0	64	89.062
	River bank	4	0	51	0	0	0	55	92.727
	Grassland	0	0	0	48	4	2	54	88.888
	Shrubland	0	3	0	9	53	3	68	77.941
	Woodland	0	0	0	0	3	55	58	94.827
**Grand Total:**		**60**	**60**	**60**	**60**	**60**	**60**	**360**	
**Producer's Accuracy:**		91.666	95	85	80	88.333	91.666		
**Samples: 360 Overall Accuracy: 88.61% Kappa Statistic: 86.30%**									

Kappa Coefficient
Number of observed agreements: 319 (88.61% of the observations)

Number of agreements expected by chance: 60.0 (16.67% of the observations)

Kappa= 0.863

SE of kappa = 0.020

95% confidence interval: From 0.824 to 0.903

The strength of agreement is considered to be 'very good'.

The calculation of weighted kappa, below, assumes the categories are ordered and accounts for how far apart the two raters are. This calculation uses linear weights.

Weighted Kappa= 0.912

(B) Year 2008.

		Land cover classification for GNP							
		**Contingency Matrix**							
		**Observed land cover Classes**						**Grand Total:**	**User's Accuracy :**
		**Water**	**Bare land**	**River bank**	**Grassland**	**Shrub land**	**Woodland**		
**Mapped land cover Classes:**	**Water**	49	0	6	0	0	0	61	81.667
	**Bare land**	0	52	3	0	0	0	64	86.667
	**River bank**	9	0	51	0	0	0	55	85
	**Grassland**	0	2	0	45	12	4	54	75
	**Shrubland**	2	6	0	11	44	3	68	73.333
	**Woodland**	0	0	0	4	4	53	58	88.333
**Grand Total:**		**60**	**60**	**60**	**60**	**60**	**60**	**360**	
**Producer's Accuracy:**		89.091	94.545	85	71.429	66.667	86.885		
		**Samples: 360 Overall Accuracy: 81.667% Kappa Statistic: 0.78**							

Kappa Coefficient

Number of observed agreements: 294 (81.67% of the observations)

Number of agreements expected by chance: 60.0 (16.67% of the observations)

Kappa= 0.780

SE of kappa = 0.024

95% confidence interval: From 0.732 to 0.828

The strength of agreement is considered to be 'good'.

Weighted Kappa= 0.843

(C) Year 1998.

**Land cover classification for GNP**									
**Contingency Matrix**									
		**Observed land cover Classes**						**Grand Total:**	**User's Accuracy:**
		**Water**	**Bare land**	**River bank**	**Grassland**	**Shrub land**	**Woodland**		
**Mapped land cover Classes:**	**Water**	56	0	2	0	0	0	61	94.915
	**Bare land**	0	48	4	3	0	0	64	80
	**River bank**	4	3	54	0	0	0	55	90
	**Grassland**	0	2	0	55	4	0	54	91.667
	**Shrubland**	0	7	0	4	53	3	68	88.333
	**Woodland**	0	0	0	1	3	57	58	95
**Grand Total:**		**60**	**60**	**60**	**60**	**60**	**60**	**360**	
**Producer's Accuracy:**		96.522	92.308	90	90.164	79.104	93.443		
**Samples: 360 Overall Accuracy: 89.972% Kappa Statistic: 0.88**									

Kappa Coefficient

Number of observed agreements: 323 (89.97% of the observations)

Number of agreements expected by chance: 59.8 (16.67% of the observations)

Kappa= 0.880

SE of kappa = 0.019

95% confidence interval: From 0.842 to 0.917

The strength of agreement is considered to be 'very good'.

Weighted Kappa= 0.917
