# Supplementary material for: Changing landscape configuration demands ecological planning: Retrospect and prospect for megaherbivores of North Bengal
Source: PLoS One. 2019 Dec 19;14(12):e0225398. doi: 10.1371/journal.pone.0225398 (PMC6922392; doi:10.1371/journal.pone.0225398)
Supplement: S3 Table — (PDF) [file pone.0225398.s003.pdf]

S3 Table. Representing the transition in hectors from 1998-2008 and from 2008-2018.

Change	Area (ha) (1998-2008)	Area (ha) (2008-2018)
Water to Water	173.07	119.52
Water to River bank	148.95	173.88
Water to Bare land	71.82	153
Water to Woodland	0.9	0.27
Water to Grassland	7.92	75.06
Water to Shrubland	103.5	16.47
River bank to Water	166.32	70.47
River bank to River bank	187.92	146.34
River bank to Bare land	163.8	202.86
River bank to Grassland	3.15	26.1
River bank to Shrubland	63.54	3.78
Bare land to Water	90.36	9.27
Bare land to River bank	71.46	13.59
Bare land to Bare land	250.47	357.57
Bare land to Woodland	0.27	4.32
Bare land to Grassland	7.56	208.62
Bare land to Shrubland	252.18	207.63
Woodland to Water	10.8	2.16
Woodland to River bank	1.62	1.44
Woodland to Bare land	2.7	0.27
Woodland to Woodland	4180.59	5079.6
Woodland to Grassland	355.32	24.75
Woodland to Shrubland	7.11	218.07
Grassland to Water	47.61	10.98
Grassland to River bank	17.01	10.8
Grassland to Bare land	20.43	6.03
Grassland to Woodland	886.14	555.57
Grassland to Grassland	526.77	206.91
Grassland Shrubland	217.62	328.95
Shrubland to Water	50.04	42.84
Shrubland to River bank	22.59	52.92
Shrubland to Bare land	291.78	266.4
Shrubland to Woodland	258.39	4.5
Shrubland to Grassland	218.52	210.96
Shrubland to Shrubland	211.68	278.01
