# Supplementary material for: Changing landscape configuration demands ecological planning: Retrospect and prospect for megaherbivores of North Bengal
Source: PLoS One. 2019 Dec 19;14(12):e0225398. doi: 10.1371/journal.pone.0225398 (PMC6922392; doi:10.1371/journal.pone.0225398)
Supplement: S4 Table — (PDF) [file pone.0225398.s004.pdf]

S4 Table. Representing the available population and demographic data for megaherbivores of Gorumara and North Bengal. [Source: West Bengal wildlife wing: https://www.wildbengal.com].

Rhino population in Gorumara

Year	Rhino count in Gorumara
**1969**	12
**1974**	6
**1978**	8
**1986**	8
**1989**	12
**1993**	12
**1996**	14
**1997**	14
**1999**	19
**2000**	19
**2002**	22
**2004**	25
**2006**	27
**2008**	31
**2011**	35
**2012**	43
**2014**	50
**2015**	49
**2019**	50+ [Preliminary report]

Rhino population (as per census 2015)

**Category**	Rhino No.
Male	22
Female	17
Sex undefined	2
Calf	8
**Total**	**49**

Elephant population in North Bengal

**Year**	**Elephant No.**
2002	292
2005	350
2007	350
2010	529
2014	590
2017	488

Gaur population in North Bengal

**Year**	**Gaur No.**
1989	240
1993	425
1997	550
1998	530-560
2002	1180-1284
2009-2010	901 + in Gorumara and adjoining areas only
2012-13	414 in Buxa tiger reserve of North Bengal only
2013-2014	782 in Buxa tiger reserve of North Bengal only
