# Supplementary material for: Changing landscape configuration demands ecological planning: Retrospect and prospect for megaherbivores of North Bengal
Source: PLoS One. 2019 Dec 19;14(12):e0225398. doi: 10.1371/journal.pone.0225398 (PMC6922392; doi:10.1371/journal.pone.0225398)

S1 Fig. Landcover transition map of GNP. A. indicating the transition types between 1998 and 2008**, B.** indicating the transition types between 1998 and 2008**.**

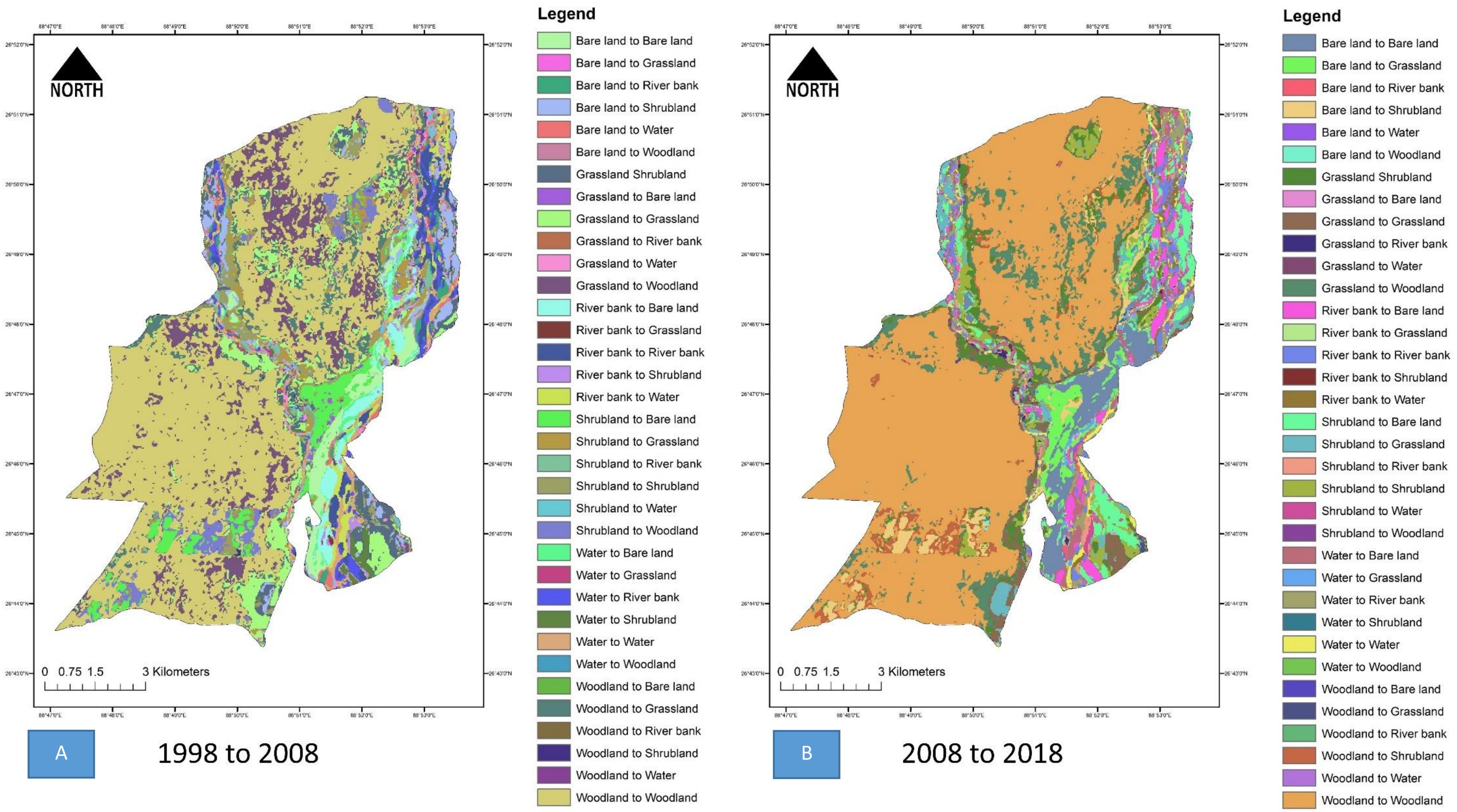

Supplement: S1 Fig — A. indicating the transition types between 1998 and 2008, B. indicating the transition types between 1998 and 2008. (PDF) [file pone.0225398.s005.pdf]
