# Supplementary material for: Changing landscape configuration demands ecological planning: Retrospect and prospect for megaherbivores of North Bengal
Source: PLoS One. 2019 Dec 19;14(12):e0225398. doi: 10.1371/journal.pone.0225398 (PMC6922392; doi:10.1371/journal.pone.0225398)

S2 Fig. Showing the frequency distribution, respective raster visualization, spatial distribution of megaherbivores variable and response curve of (A) Anthropogenic and topographic variables (B) Grassland variables, (C) Woodland variables, (D) Shrubland variables and (E) Landscape level variables.

(A) Anthropogenic and topographic variables. Where a=Euclidian distance from road, b= Compound topographic index, c= SRTM DEM data acquired from USGS and d= Euclidian distance from road.

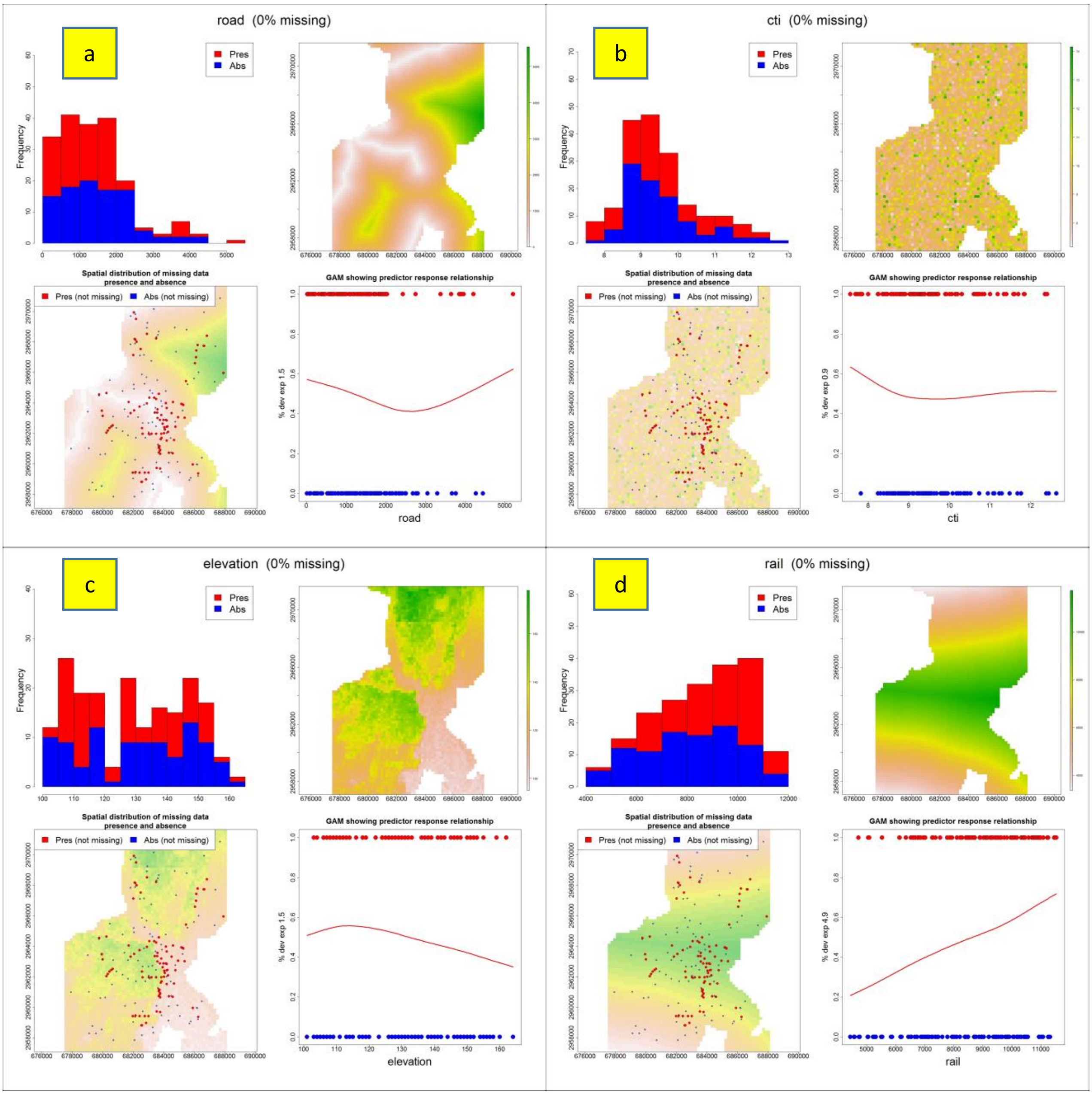

(B). Grassland variables. Where a=Euclidian distance, b=Patch density, c= Area-weighted mean patch area, d= Interspersion and juxtaposition index, e= Largest patch index and f= Number of patches.

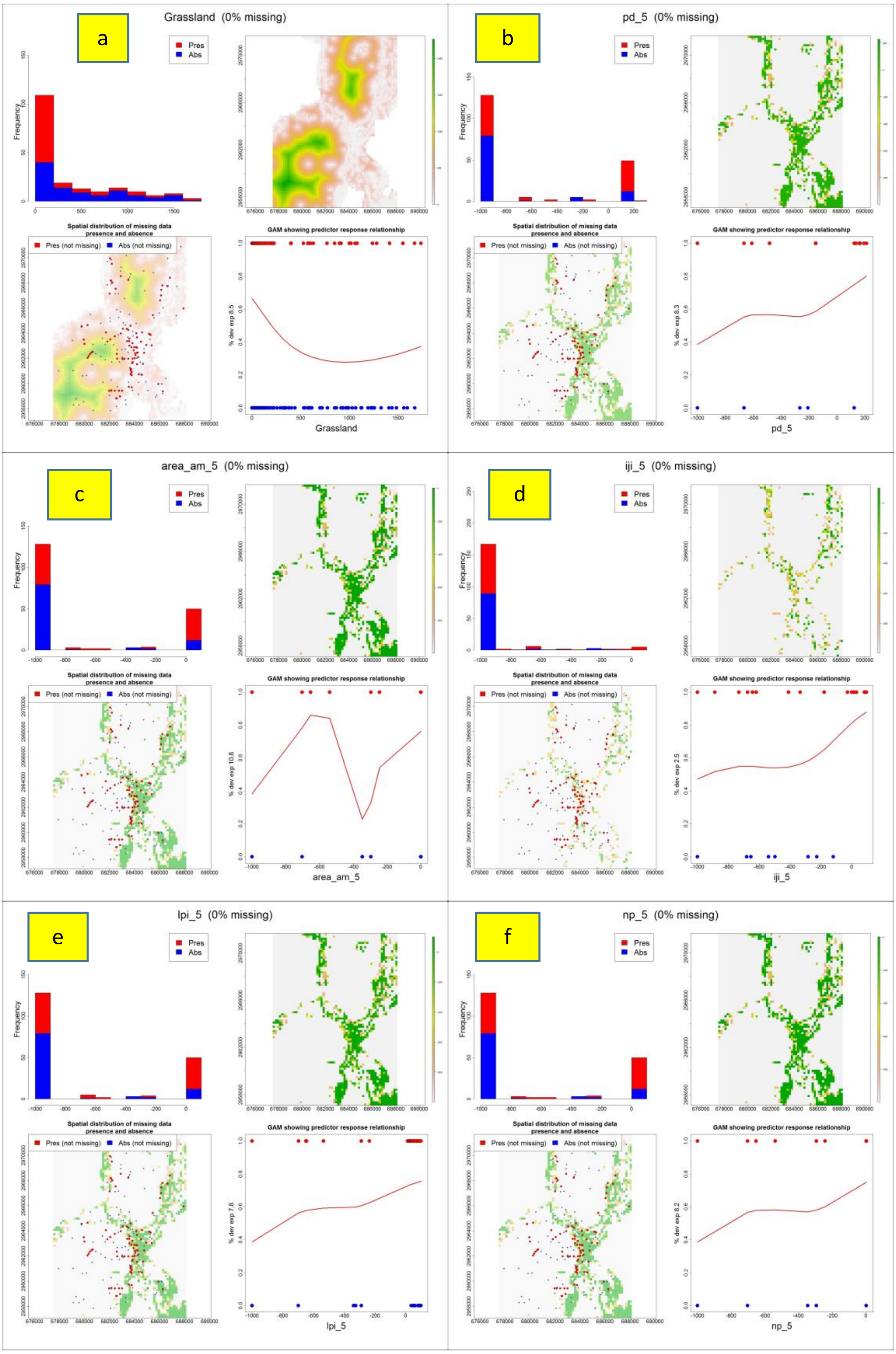

(C). Woodland variables. Where a=Euclidian distance, b= Interspersion and juxtaposition index, c= Largest patch index, d=Number of patches, e= Patch density and f= Area-weighted mean patch area.

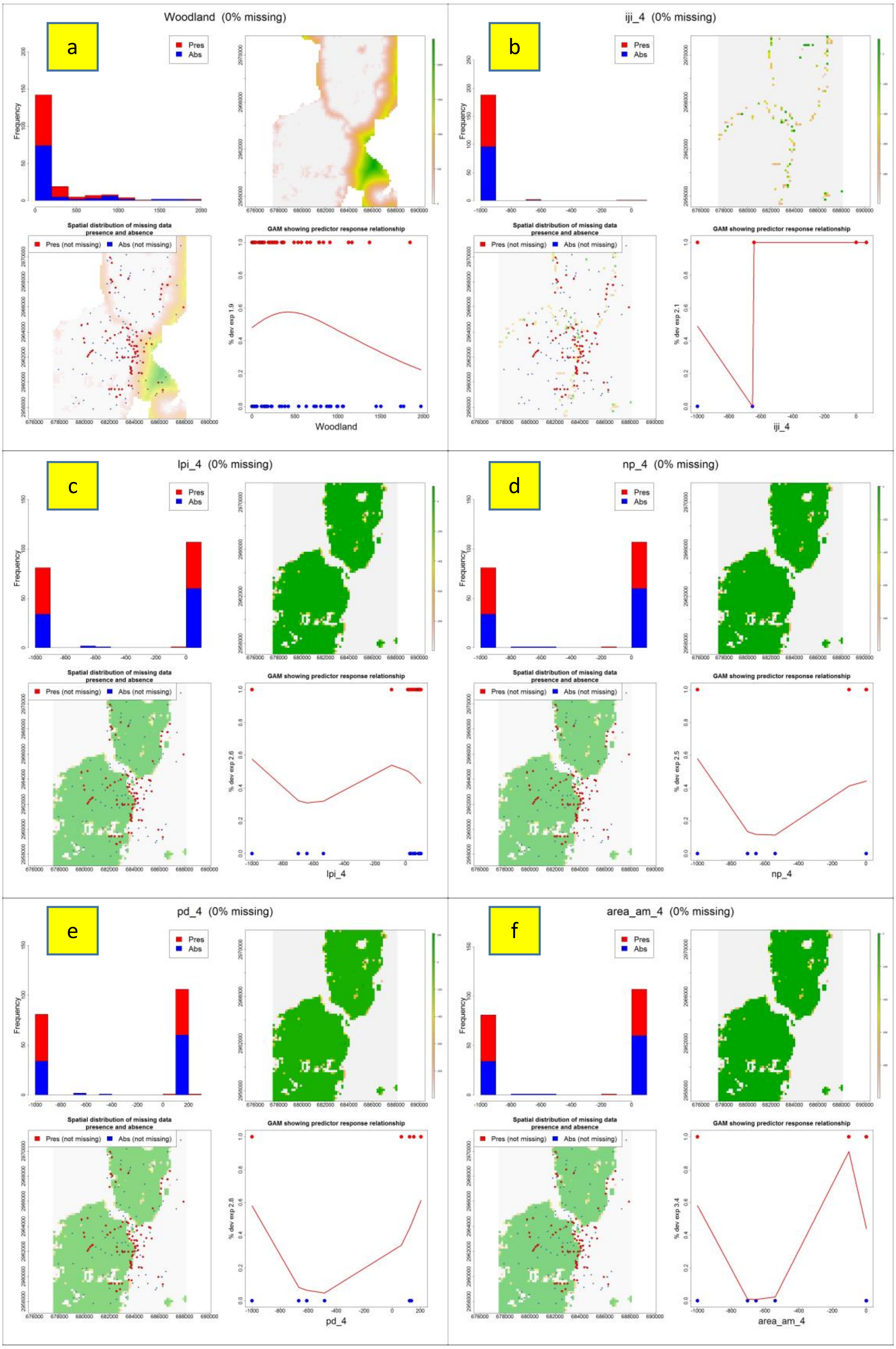

(D). Shrubland variables. Where a=Euclidian distance, b= Area-weighted mean patch area, c= Interspersion and juxtaposition index, d= Largest patch index, e= Number of patches and f= Patch density.

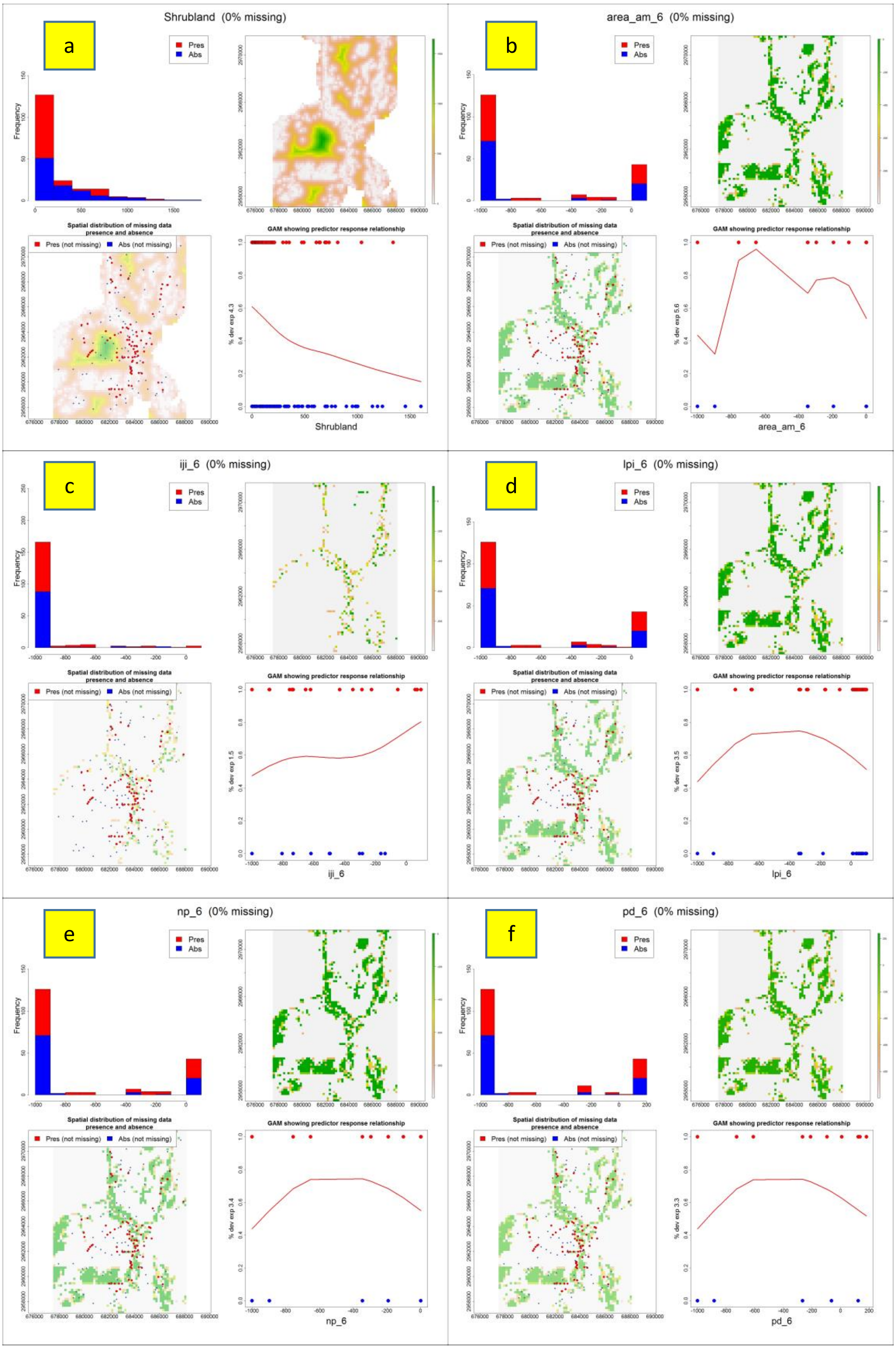

(E). Landscape level variables. Where a= Interspersion and juxtaposition index, b= Patch density, c= Shannon's diversity index, d= Simpson's evenness index, e= Aggregation index and f= Edge density.

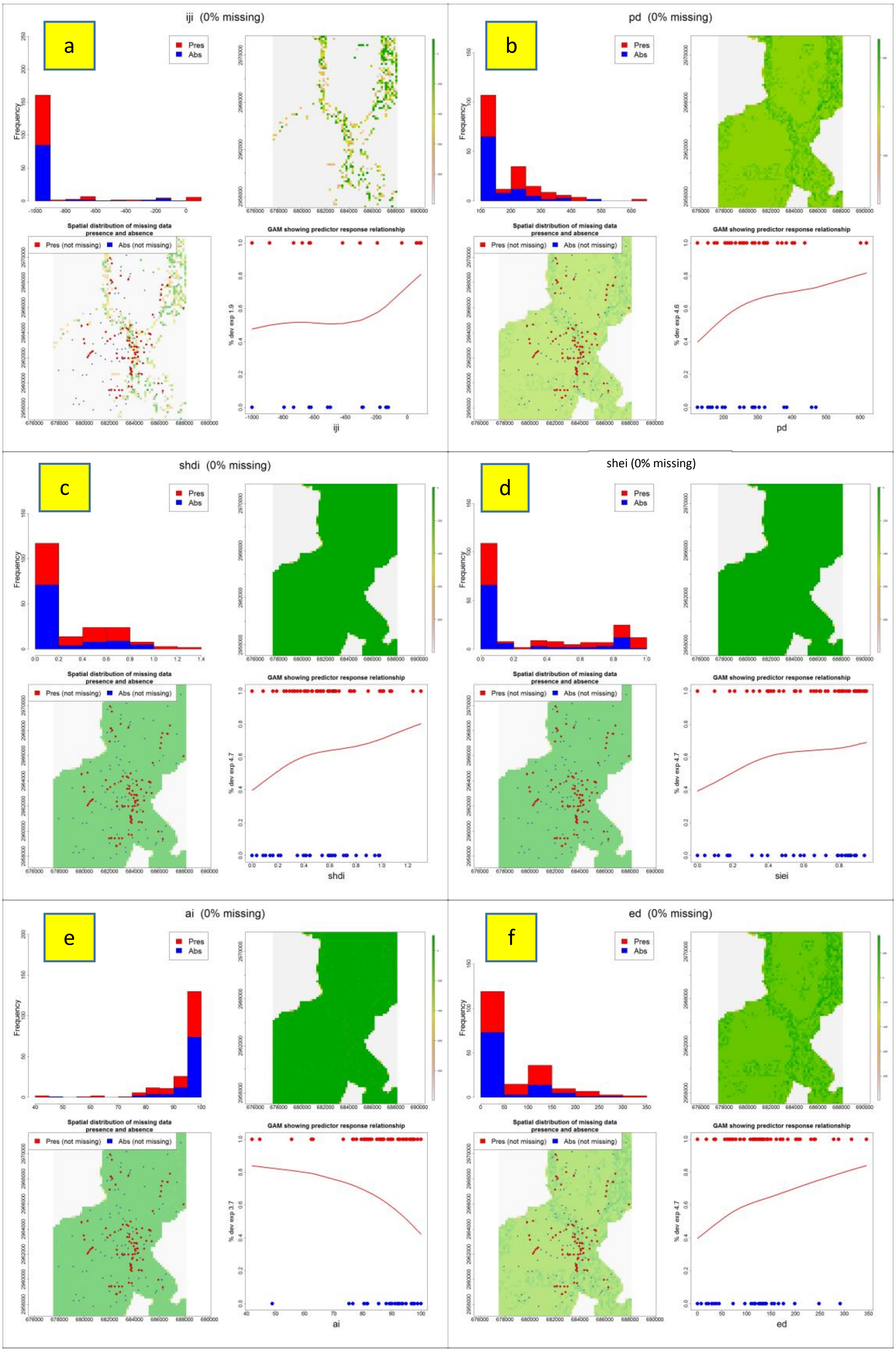

Supplement: S2 Fig — Showing the frequency distribution, respective raster visualization, spatial distribution of megaherbivores variable and response curve of (A) Anthropogenic and topographic variables (B) Grassland variables, (C) Woodland variables, (D) Shrubland variables and (E) Landscape level variables. (PDF) [file pone.0225398.s006.pdf]
