# Supplementary material for: Changing landscape configuration demands ecological planning: Retrospect and prospect for megaherbivores of North Bengal
Source: PLoS One. 2019 Dec 19;14(12):e0225398. doi: 10.1371/journal.pone.0225398 (PMC6922392; doi:10.1371/journal.pone.0225398)

S3 Fig. Bar graph representing percentage of deviation explained by all covariates from Generalized Additive Modeling using the Software for Assisted Habitat Modeling (SAHM).

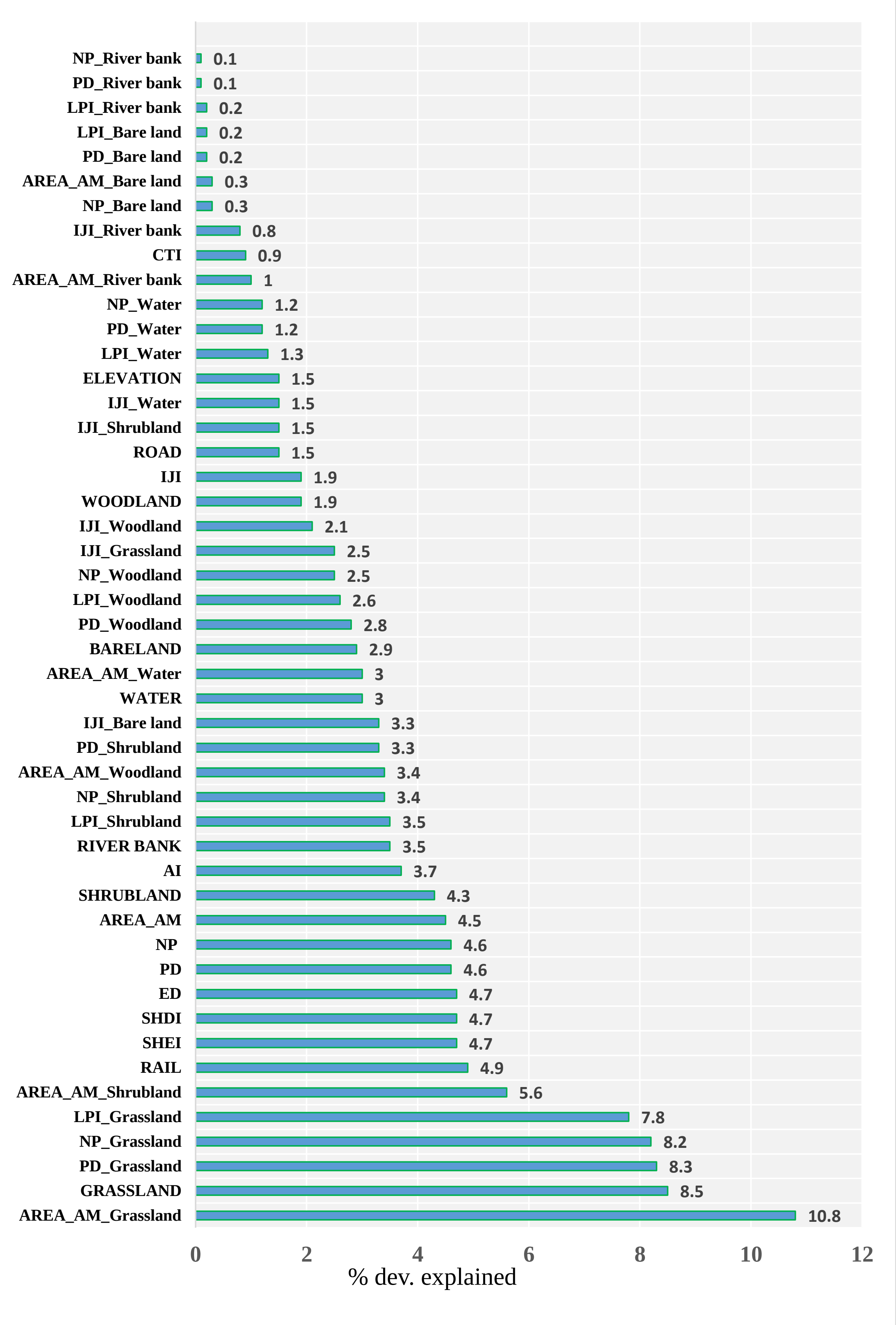

Supplement: S3 Fig — (PDF) [file pone.0225398.s007.pdf]
