# Supplementary figures and images for: Changing landscape configuration demands ecological planning: Retrospect and prospect for megaherbivores of North Bengal
Source: PLoS One. 2019 Dec 19;14(12):e0225398. doi: 10.1371/journal.pone.0225398 (PMC6922392; doi:10.1371/journal.pone.0225398)

S4 Fig. Methodological flow chart of the study

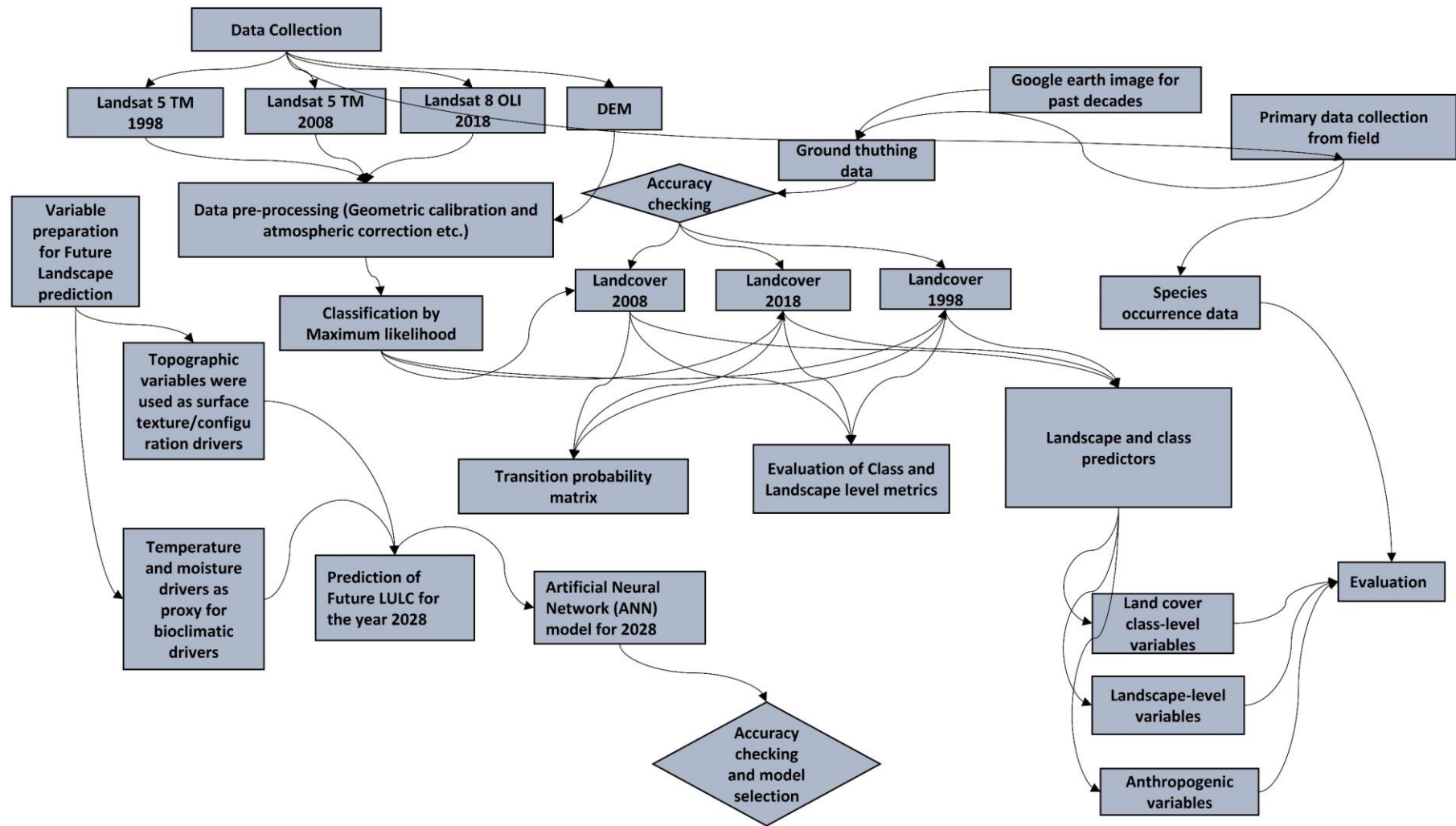

Supplement: S4 Fig — (PDF) [file pone.0225398.s008.pdf]
